# Supplementary material for: Integrin-alpha-6+ Candidate stem cells are responsible for whole body regeneration in the invertebrate chordate Botrylloides diegensis
Source: Nat Commun. 2020 Sep 7;11:4435. doi: 10.1038/s41467-020-18288-w (PMC7477574; doi:10.1038/s41467-020-18288-w)
Supplement: Supplementary file 1 — Supplementary Information [file 41467_2020_18288_MOESM1_ESM.pdf]

Integrin-alpha-6+ Candidate Stem Cells are responsible for whole body regeneration in an invertebrate chordate

Kassmer et al

Supplementary Figures:

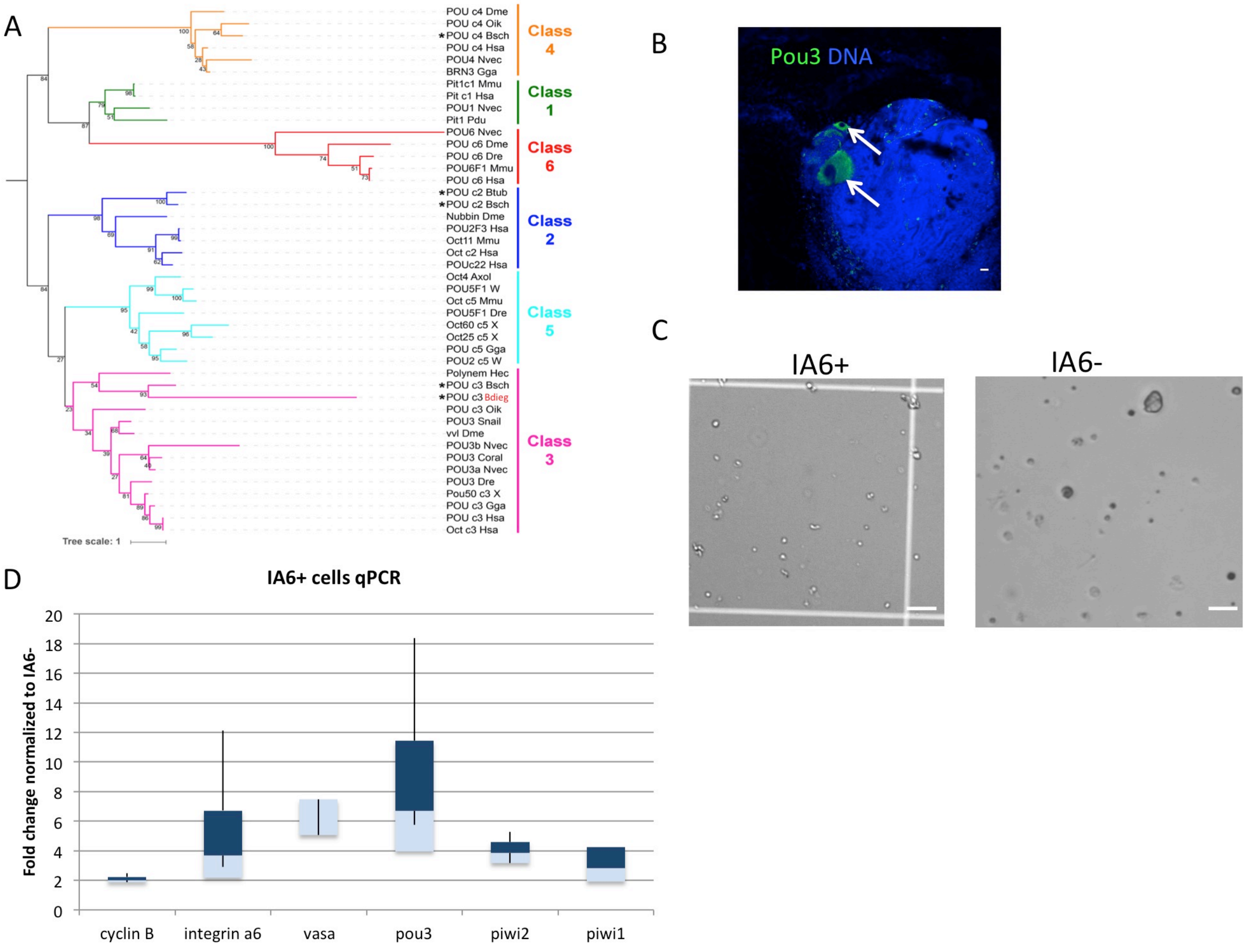

Supplementary Figure 1: Phylogenetic analysis of pou proteins, expression of pou3 on whole animals, expression levels of integrin-alpha6, vasa, pou3, piwi1 and piw2 in IA6+ cells, images of IA6+ and IA6- cells. A: Maximum likelihood phylogenetic analysis of POU family members. Phylogenetic tree was constructed with RAxML using a maximum likelihood method, the JTT substitution matrix, and empirical frequencies. Botryllid POU proteins identified in this study are marked by asterisks. Families of POU proteins are labeled and indicated by vertical lines. Nodes are labeled with bootstrap values in units of percentage. The scale bar for branch lengths indicates the mean number of inferred substitutions per site. B: FISH for pou3 (green) on whole colonies. Pou3 is expressed in germ cells associated with the secondary bud (arrows). Images are representatives of 4 independent experiments. Scale bar: 20µm. C: Brightfield images of FACS-sorted IA6+ and IA6- cells. Images are representatives of 4 independent experiments. Scale bar 10µm. D: quantitative-real-time-PCR analysis of gene expression in FACS-sorted IA6+ cells. Data from 4 independent experiments are shown as normalized to IA6- cells. Box plot shows interquartile range (the distance between the upper and lower quartiles) and whiskers indicate the minimum and maximum of the data. Source data are provided as a Source Data file.

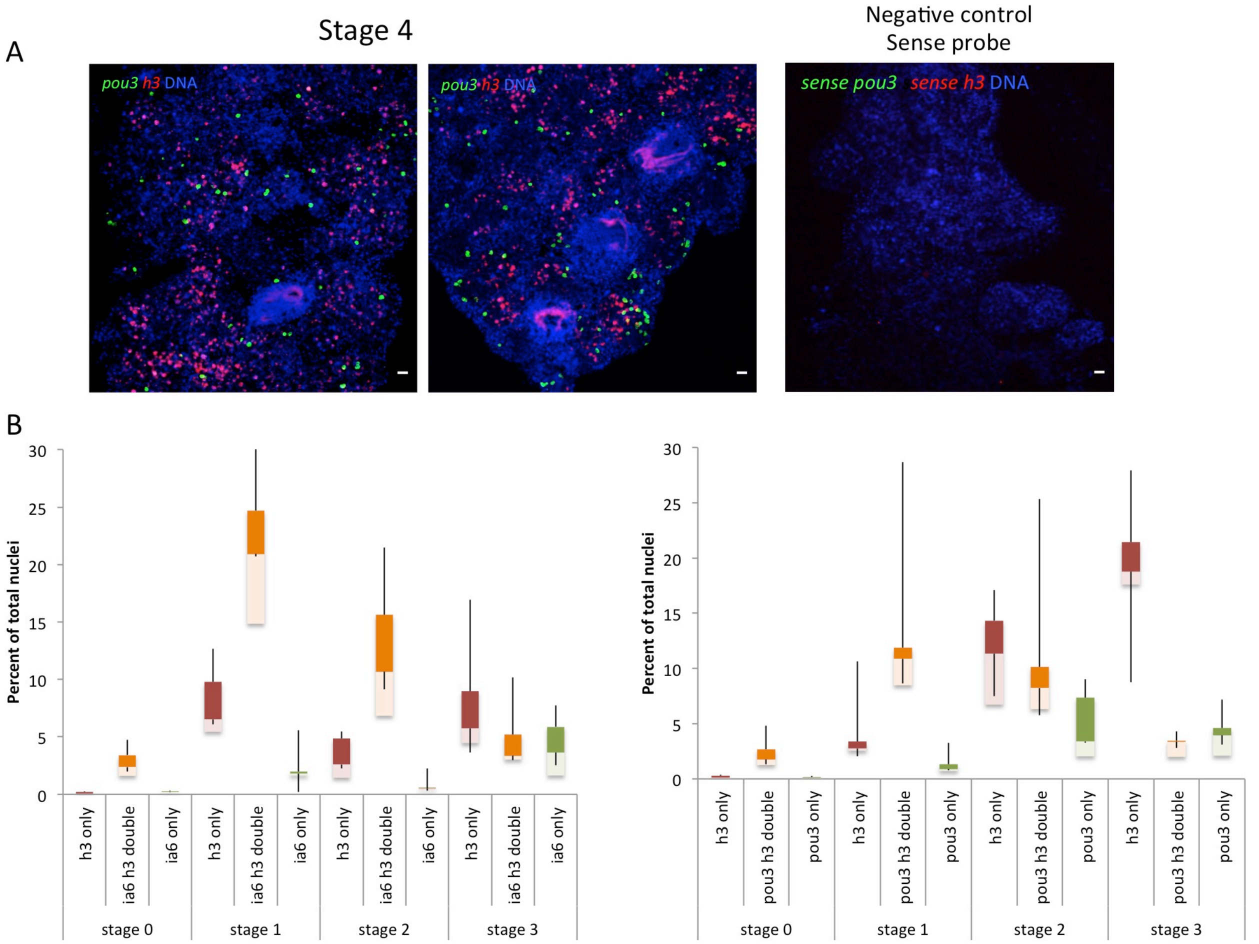

Supplementary Figure 2: Expression pattern of *pou3* and *histone 3* during stage 4 of WBR, and numbers of ia6/h3 and *pou3/h3* positive cells during stages 0 to 3. A) FISH showing expression of *pou3* (green) and *histone 3* (h3, red) during stage 4of WBR. DNA was stained with Hoechst (blue). Images are representatives of 4 independent experiments Scale bars 20μm. Negative control shows signal with sense probe. B) Single positive (*pou3, ia6* or *h3*) and double positive (*pou3/h3* or *ia6/h3*) cells were counted using the cell counter feature in FIJI, and for each stage, images from 4 independent samples were counted. Graphs show percentages of positive cells among all Hoechst-positive nuclei per time point. Box plot shows interquartile range (the distance between the upper and lower quartiles) and whiskers indicate the minimum and maximum of the data. Source data are provided as a Source Data file.

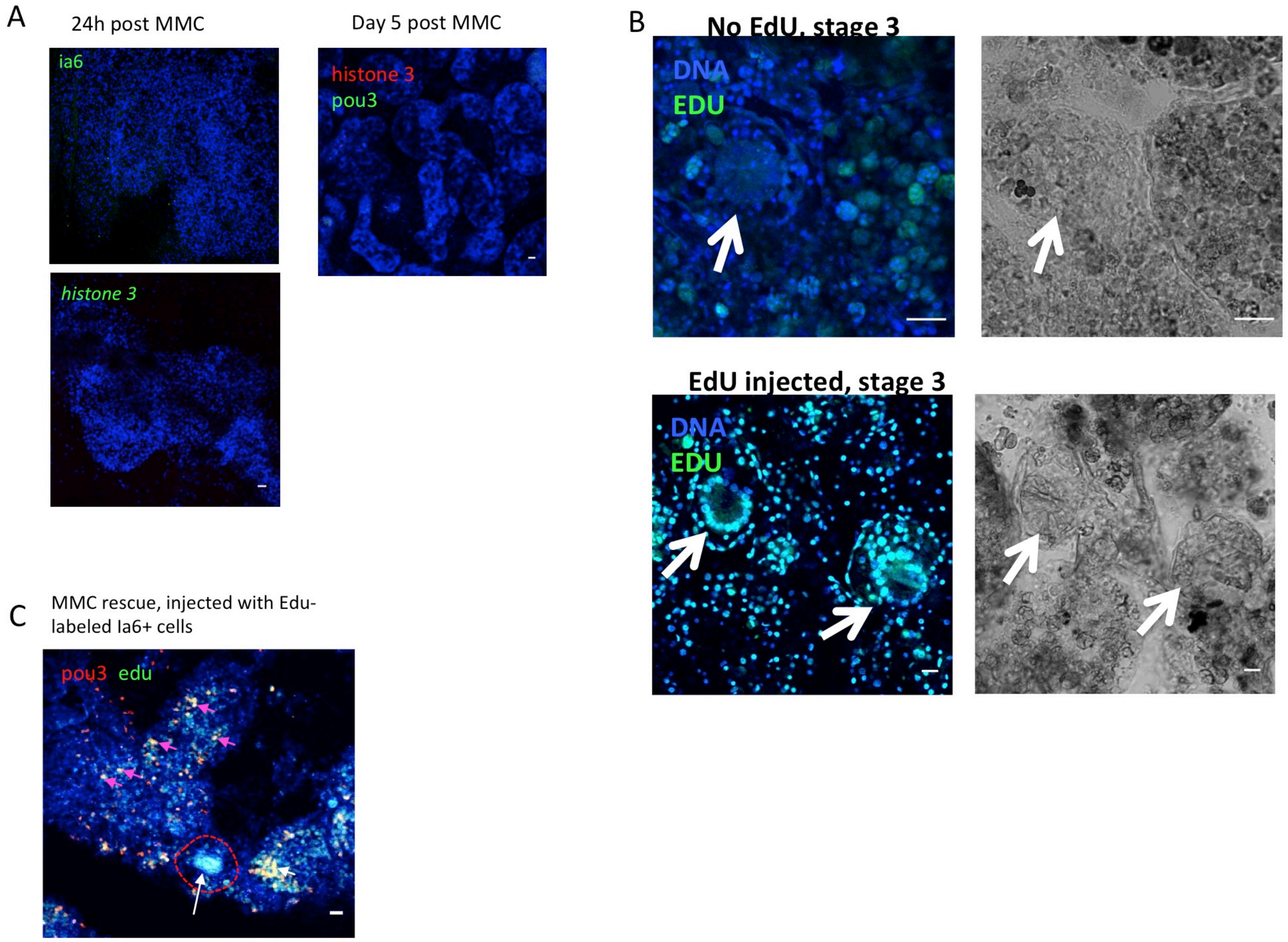

Supplementary Figure 3: Removal of *pou3/ia6/h3* positive cells after Mitomycin C treatment, Edu staining negative and positive controls, and *pou3* expression in Edu-labeled cells after transplantation of Edu+ IA6+ cells. A: FISH for ia6 and histone 3 24h after treatment with Mitomycin C. FISH for histone 3 and pou3 5 days after treatment with Mitomycin C. Images are representatives of 4 independent experiments. Scale bar 20µm. B: Overlay of nuclear staining (Hoechst, blue) and Edu (green). In vessel sections from stage 3 samples not injected with EdU, no green EdU signal is detected. In stage 3 samples that have been injected with EdU at stage 0, EdU-positive cells are present in the blood as well as in regenerating double vesicles (white arrows). Images on the right show brightfield. Double vesicles are clearly visible (white arrows). Images are representatives of 4 independent experiments. Scale bars 20µm. C: MMC treated sample injected with Edu-labeled IA6+ cells. The sample was fixed at stage 3 (white arrow and red outline indicate double vesicle) and FISH for ia6 was combined with Edu- staining. Edu-positive ia6+ cells are visible in the blood (pink arrows). Images are representatives of 4 independent experiments. Scale bar 20µm.

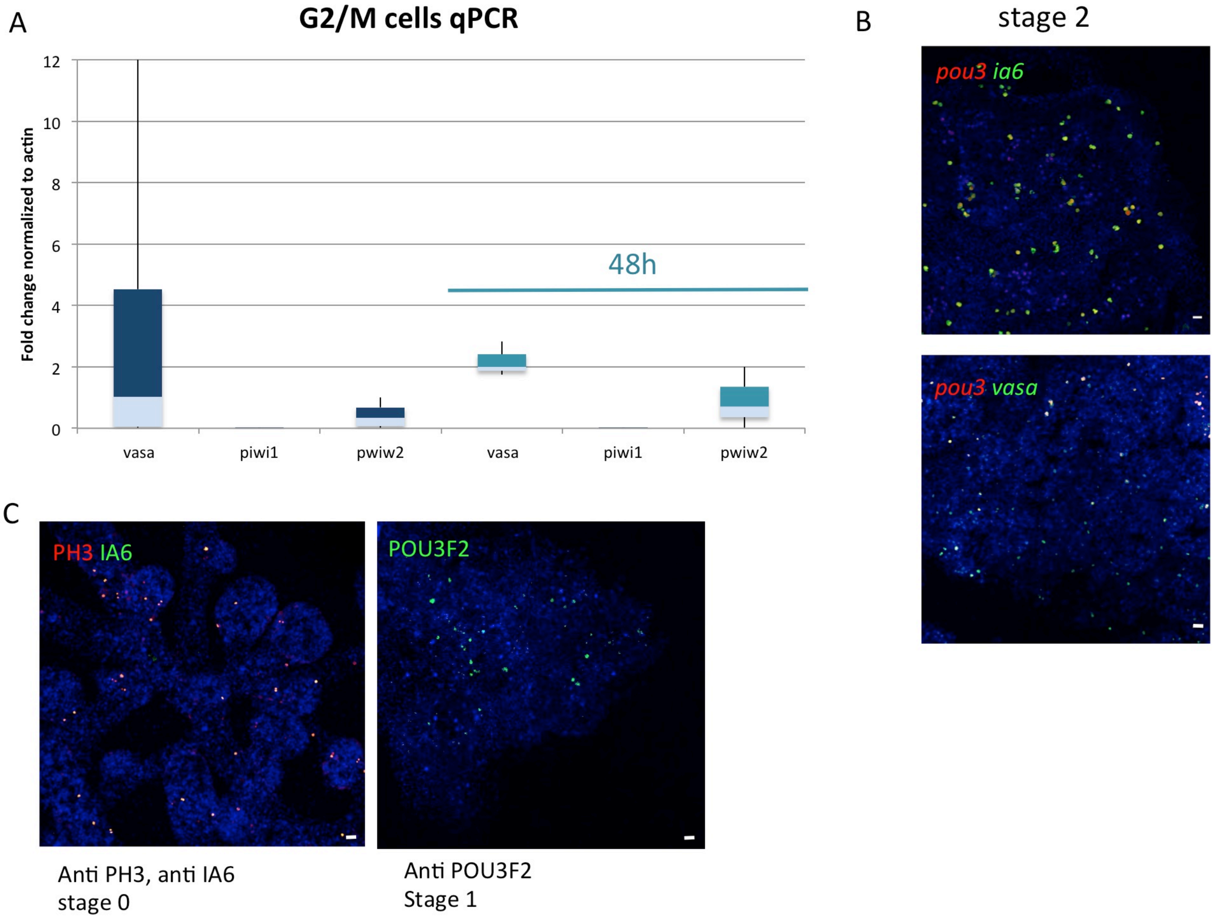

Supplementary Figure 4:Expression of vasa, piwi1 and piw2 in G2/M cells at 0h and 48h, co-expression of pou3/ia6 and pou3/vasa during stage 3, and expression of IA6 and POU3 protein. A: Q-PCR analysis of gene expression in cycling G2/M cells at different time points of WBR. Data from 4 independent experiments are shown as normalized to actin. Box plot shows interquartile range (the distance between the upper and lower quartiles) and whiskers indicate the minimum and maximum of the data. Source data are provided as a Source Data file. B: FISH showing co-expression of *pou3* (red) and *vasa* (green) in stage 2. DNA was stained with Hoechst (blue). Images are representatives of 4 independent experiments. Scale bar 20µm. C: Immunofluorescence on cryosections of stage 0 vessel fragments. Left: Anti-Integrin-alpha 6 (green) and Anti-Histone H3 (phospho S10) (red). Right: Anti POU3F2 (green). Images are representatives of 4 independent experiments. Scale bar 20µm.

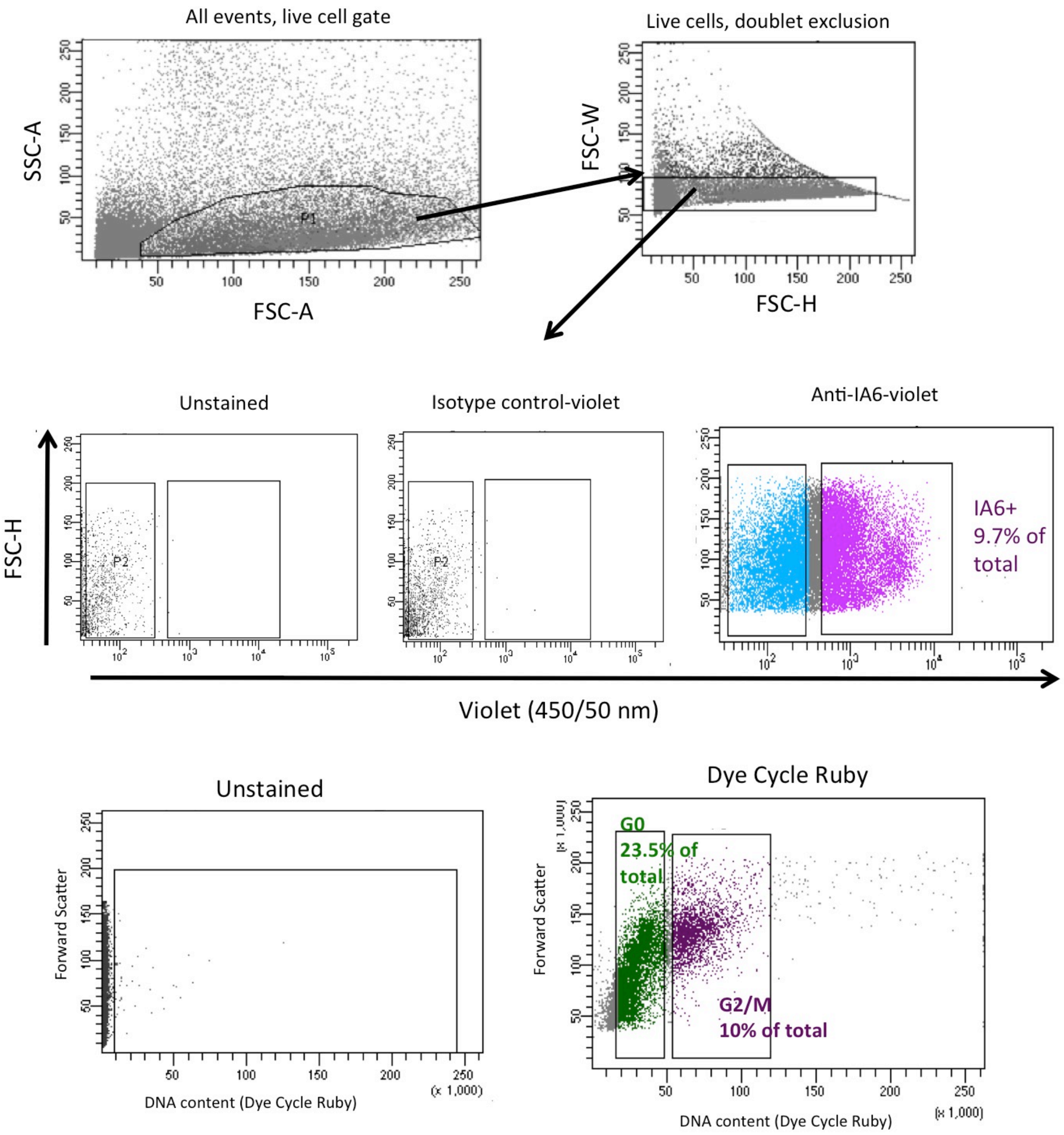

Supplementary Figure 5: **Fluorescence-activated cell sorting (FACS) gating strategy.** Live cells (P1) were gated based on forward scatter (FSC) and side scatter (SSC) properties (top row). Doublets were excluded by plotting FSC-signal height (FSC-H) against FSC-signal width (FSC-W). The violet channel was chosen for staining of Integrin-alpha-6 because of very low autofluorescence, as shown in the unstained (natural fluorescent background) samples. IA6-negative cells were gated based on isotype-control staining, with 0.1% of cells in the positive gate in the isotype control sample. IA6+ cells comprised 9.7% of total cells in the sample stained with Anti-Integrin-alpha-6-violet. For Cell cycle/DNA content analysis, live cells (P1) were gated based on forward scatter (FSC) and side scatter (SSC) properties (top row). Doublets were excluded by plotting FSC-signal height (FSC-H) against FSC-signal width (FSC-W). DNA content was analyzed on a linear scale using Dye Cycle Ruby. G2/M cells have double the fluorescence intensity (DNA content) than G0 cells on a linear scale. Plots are representatives of 20 independent experiments.

Supplementary Methods:

Alignment of human Integrin-alpha-6 protein with translated *B. diegensis* integrin alpha 6 mRNA:

B. diegensis *integrin-alpha-6* mRNA sequence in Supplementary Data 2

```
tBlastn
Query: human Integrin alpha 6 protein
Subject B. diegensis integrin alpha 6 mRNA

Score:506 bits(1302), Expect:2e-160,
Method:Compositional matrix adjust.,
Identities:376/1102(34%), Positives:553/1102(50%), Gaps:114/1102(10%)

Query  24    FNLDTREDNVIRKYGDPGSLFGFSLAMHW---QLQPEDKRLLLVGAPRAEALPLQRANRT  80
             FN+D R    I K G PGSLFG S+A H    ++Q +D  LL+VGAP   A       ++
Sbjct  346   FNIDERHP--IVKTGPPGSLFGLSVAEHHVNERIQSKDNALLIVGAPTGVAYGSSDNTQS  519

Query  81    GGLYSCDITARGPCTRIEFDNDADPTSESKEDQWMGVTVQSQGPGGKVVTCAHRYEKRQH  140
             G +Y C + +   CT +          E+   QW+GV+V+S  PGG+++TCAHRY
Sbjct  520   GAVYKCSVFSDANCTLVPIIPPNSGRVENTTSQWLGVSVKSHKPGGQILTCAHRYTL---  690

Query  141   VNTKQESRDIFGRCYVLSQNLRIEDDMDGGDWSFCDGRLRG-----HEKFGSCQQGVAAT  195
                  +     GRC++L ++     D    ++  C+ +  G     HE +G CQ G +
Sbjct  691   --VGSDWVAPVGRCFMLEKDTTPVQDEFSAEYVPCEDKQDGLGRYSHEGYGYCQAGASVN  864

Query  196   FTK------DFHYIVFGAPGTYNWKGIVRVEQKNNTFFDMNIFEDGPYEVGGETEHDESL  249
             F           Y++ GAPG+ +W G V   +K   F             G   E   S
Sbjct  865   FADVPASDDGESYVLIGAPGSIHWSGAVLATRKGGDF-------------GLSVEKVWSD  1005

Query  250   VPVPANSY-LGFSLDSGKGIVSKDEITFVSGAPRANHSGAVVLLKRDMKS-------AHL  301
               +   +Y +G S+ +G  I  +D + FV+GAP AN +GAV +L++           ++L
Sbjct  1006  KDLTMTNYQMGSSVLAGF-IYREDAVNFVTGAPGANTTGAVYILEKTTPDVTNSDGDSYL  1182

Query  302   LPEHIFDGEGLASSFGYDVAVVDLNKDGWQDIVIGAPQYFDRDGEVGGAVYVYMNQQGRW  361
                   +G+ +AS FG+D+ ++D+  DG  D++IGAPQ++DR+ +VGGAVYVY+N+
Sbjct  1183  RIVETVNGDKVASRFGHDILLLDVTGDGKLDLIIGAPQFYDRNDQVGGAVYVYVNKGLST  1362

Query  362   NNVKPI-RLNGTKDSMFGIAVKNIGDINQDGYPDIAVGAPYDD--LGKVFIYHGSAN---  415
                 P  RL G  DS FG+AV + GD+N DG  DIA+GAP  +   G V+IYHG ++
Sbjct  1363  IGPSPTQRLFGNIDSYFGMAVASAGDVNMDGVNDIAIGAPGGNKWTGVVYIYHGDSSADG  1542

Query  416   -GINTKPTQV--------LKGISPYFGYSIAGNMDLDRNSYPDVAVGSLSDSVTIFRSRP  466
              G+  KP+Q+        L G S   GYS++G +D+D N YPD+AVGSLSDS  +F SRP
Sbjct  1543  MGVGAKPSQIIEASKISALGGNSYGLGYSLSGGLDMDLNGYPDIAVGSLSDSAVVFFSRP  1722

Query  467   VINIQKTITVTPNRIDLRQKTACGAPSGICLQVKSCFEYTANPAGYNPSISIVGTLEAEK  526
             V+N+  TIT    +I+L +      PS   L ++ C  YTA P  ++  + + G +E +
Sbjct  1723  VVNVTGTITGPNKKIELSED-----PSEQVLDIEICLRYTALPPSFDERVRVEGYVELDS  1887

Query  527   ERRKSGLSSRVQFRNQGSEPKYTQE-----LTLKRQ--KQKVCMEETLWLQDNIRDKLRP  579
              R + GL SR+ F+   ++    Q      +TL RQ  ++  C     +++++IRDKL P
Sbjct  1888  GRVERGLLSRLSFKRSSTKESEGQSKKPFVMTLHRQSAQRDKCDTYKTYMKNDIRDKLTP  2067

Query  580   IPI-----TASVEIQEPSSRRRVNSLPEVLPILNSDEPKTAHIDVHFLKEGCGDDNVCNS  634
             I +     T +   ++   RR     P + PI+N+    TA  ++ F K  CGDD +C+S
Sbjct  2068  IDLKLTFTTPNDPPKKRRRRRDTTYTPPI-PIMNTAITDTAVAEIEFAKR-CGDDEICSS  2241

Query  635   NL-KLEYKFCTREGNQDKFSYLPIQKGVPELVLKDQKDIALEITVTNSPSNPRNPTKDGD  693
             NL K  Y    + GN    +    + G P L+L  ++ I L I VTN         ++G+
Sbjct  2242  NLEKKAYYQVLKNGNWSDLTRK--RNGQPLLILGTEEQIGLVIEVTN---------QNGE  2388

Query  694   DAHEAKLIATFPDTLTYSAYRELRAFPEKQLSCVAN-QNGSQADCELGNPFKRNSNVTFY  752
             DAH+A++    PD +    YR +       + C  + +N S   C LGNPFK  S+V F
Sbjct  2389  DAHQARMNIVLPDEV---EYRRIELMSGNAIHCDPDTKNRSLVVCHLGNPFKEESSVKFK  2559

Query  753   LVLSTTEVTFDTPDLDINLKLETTSNQDNLAPITAK-AKVVIELLLSVSGVAKPSQVYFG  811
             L +S T+         + L+L TTS Q    P T     V +E  L + G     QV F
```

```
             [illegible]
Sbjct  2560  LKMSKTQSIQKATQFAVKLQLLTTSQQKIPDPGTEYLVYVEVEAQLELVGYPDREQVDFS  2739

Query  812   GTVVGEQAMKSEDEVGSLIEYEFRVINLGKPLTNLGTATLNIQWPKEISNGKWLLYLVKV  871
             G V+GE A+    + G+L  +++ + N G  +       L I WP+ I NGKWL YL+K
Sbjct  2740  GVVIGESAVMKPTDAGTLHMHKYEIKNSGTGIVE--DVALKIMWPQAIINGKWLFYLLKA  2913

Query  872   ESKGLEKVTCEPQKEINSLNLTESHNSRKKREITEKQIDDNRKFSLFAERKYQTLNC--S  929
             E +G    +     +   LNL    + R KR++       N K       KY +LNC
Sbjct  2914  EVQGFGNCSSPGNVDPLQLNL---KSKRAKRDVDPPP--GNLKDGGSPIIKYASLNCRDG  3078

Query  930   VNVNCVNIRCPLRGLDSK--ASLILRSRLWNSTFLEEYSKLNYLDILMRAFIDVTAAAEN  987
               +NCV + C L  L SK  A + L + LWNSTFLEE+S  N   + + +   V+   EN
Sbjct  3079  GGLNCVEVVCFLGDLASKVTARVDLTAILWNSTFLEEFS--NVAGVYVESDAKVSILQEN  3252

Query  988   IRLPNAGTQVR---VTVFPSKTVAQYSGVPWWiilvailagilmlallVFILWKCGFFKR  1044
             I+     T  R    TV P +       + WWII VA  AG+++L  LV I+WKCGFFKR
Sbjct  3253  IKFSEESTLERSTTTTVLPLEIFVPKQPIEWWIIAVAGAAGLILLVFLVLIMWKCGFFKR  3432

Query  1045  NKKDHYDATYHKAEIHAQPSDK  1066
                      Y KA  H Q S K
Sbjct  3433  QTL----VDYQKARRHKQASKK  3486
```

Alignments of our *B. diegensis* sequences with public *B. leachii* sequences:

Alignment of *B diegensis* actin with *B. leachii* sequence from NCBI genbank:

B diegensis actin aligns with
actin, partial [*Botrylloides leachii*] 100%
Sequence ID: ABN42646.1Length: 131

Score	Expect	Method	Identities	Positives	Gaps	Frame
273 bits(699)	8e-93	Compositional matrix adjust.	131/131(100%)	131/131(100%)	0/131(0%)	+1

Alignments with *B. leachii* transcriptome on ANISEED:
(https://www.aniseed.cnrs.fr/aniseed/default/blast_search)

B. diegensis ia6 probe sequence aligns 99%
Result #1 on Boleac.CG.SB_v3.S108.g00853.01.t
Length=5047
Score = 1336 bits (723), Expect = 0.0
Identities = 731/735 (99%), Gaps = 0/735 (0%)
Strand=Plus/Plus

B. diegensis vasa probe aligns 100%:
Result #1 on Boleac.CG.SB_v3.S892.g15055.01.t
Length=1542
Score = 638 bits (345), Expect = 0.0
Identities = 345/345 (100%), Gaps = 0/345 (0%)
Strand=Plus/Plus

B. diegensis piwi1 probe aligns 99%
Result #1 on Boleac.CG.SB_v3.S686.g13121.01.t
Length=3041
Score = 2913 bits (1577), Expect = 0.0
Identities = 1598/1610 (99%), Gaps = 0/1610 (0%)
Strand=Plus/Plus

B. diegensis notch2 probe aligns 99%
Result #1 on Boleac.CG.SB_v3.S59.g12005.01.t
Length=7928
Score = 732 bits (396), Expect = 0.0
Identities = 402/405 (99%), Gaps = 0/405 (0%)
Strand=Plus/Minus

B. diegensis notch1 probe aligns 99%
Result #1 on Boleac.CG.SB_v3.S59.g12005.01.tLength=7928
Score = 1471 bits (796), Expect = 0.0
Identities = 798/800 (99%), Gaps = 0/800 (0%)
Strand=Plus/Plus
