# Supplementary material for: Integrin-alpha-6+ Candidate stem cells are responsible for whole body regeneration in the invertebrate chordate Botrylloides diegensis
Source: Nat Commun. 2020 Sep 7;11:4435. doi: 10.1038/s41467-020-18288-w (PMC7477574; doi:10.1038/s41467-020-18288-w)
Supplement: Supplementary file 3 — Reporting Summary [file 41467_2020_18288_MOESM3_ESM.pdf]

nature research

Corresponding author(s): Susannah Kassmer

Last updated by author(s): 2020 07 20

Reporting Summary

Nature Research wishes to improve the reproducibility of the work that we publish. This form provides structure for consistency and transparency in reporting. For further information on Nature Research policies, see Authors & Referees and the Editorial Policy Checklist .

Statistics

For all statistical analyses, confirm that the following items are present in the figure legend, table legend, main text, or Methods section.

n/a	Confirmed	
☐	☒	The exact sample size (*n*) for each experimental group/condition, given as a discrete number and unit of measurement
☐	☒	A statement on whether measurements were taken from distinct samples or whether the same sample was measured repeatedly
☒	☐	The statistical test(s) used AND whether they are one- or two-sided<br>*Only common tests should be described solely by name; describe more complex techniques in the Methods section.*
☒	☐	A description of all covariates tested
☒	☐	A description of any assumptions or corrections, such as tests of normality and adjustment for multiple comparisons
☐	☒	A full description of the statistical parameters including central tendency (e.g. means) or other basic estimates (e.g. regression coefficient) AND variation (e.g. standard deviation) or associated estimates of uncertainty (e.g. confidence intervals)
☒	☐	For null hypothesis testing, the test statistic (e.g. *F*, *t*, *r*) with confidence intervals, effect sizes, degrees of freedom and *P* value noted<br>*Give P values as exact values whenever suitable.*
☒	☐	For Bayesian analysis, information on the choice of priors and Markov chain Monte Carlo settings
☒	☐	For hierarchical and complex designs, identification of the appropriate level for tests and full reporting of outcomes
☒	☐	Estimates of effect sizes (e.g. Cohen's *d*, Pearson's *r*), indicating how they were calculated

Our web collection on statistics for biologists contains articles on many of the points above.

Software and code

Policy information about availability of computer code

Data collection	LightCycler® 480 Software Version 1.5 , BD FACSDiva 8.0.1, NCBI BLASTX, NCBI primer blast
Data analysis	BD FACSDiva 8.0.1, FIJI (https://fiji.sc/), ELDA (http://bioinf.wehi.edu.au/software/elda/), MEGA7 (https://www.megasoftware.net/show_eua), RAxML (https://cme.h-its.org/exelixis/web/software/raxml/) version 8.0.0, Microsoft Excel 2011, NCBI BLASTX

For manuscripts utilizing custom algorithms or software that are central to the research but not yet described in published literature, software must be made available to editors/reviewers. We strongly encourage code deposition in a community repository (e.g. GitHub). See the Nature Research guidelines for submitting code & software for further information.

Data

Policy information about availability of data

All manuscripts must include a data availability statement. This statement should provide the following information, where applicable:

- Accession codes, unique identifiers, or web links for publicly available datasets
- A list of figures that have associated raw data
- A description of any restrictions on data availability

All data generated in this study are included in the article or the supplementary files. Databases used: NCBI genbank, ANISEED Ascidian database (https://www.aniseed.cnrs.fr/)

Field-specific reporting

Please select the one below that is the best fit for your research. If you are not sure, read the appropriate sections before making your selection.

Life sciences study design

All studies must disclose on these points even when the disclosure is negative.

Sample size	Rescue experiments were performed on 16-30 biologically independent samples, to allow for the ability to calculate the frequency of ISC's capable of rescue. Gene expression studies were performed on 3-6 biologically independent samples per time point in order to be able to calculate Averages or Means and in order to be able to show data variability.
Data exclusions	No data were excluded from the analyses.
Replication	Rescue experiments were performed on 16-30 biologically independent samples in two independent experiments, both of which were successful. qPCR on sorted cells was performed on at least 3 biologically independent samples per time point, which were collected and processed on different days, and all replications were successful. FISH was performed on at least 3 independent samples that were collected and processed on different days. All replications were successful.
Randomization	Samples were randomly allocated to each group.
Blinding	The investigators were not blinded to the sample identity because the same person prepared and analyzed the samples.

Reporting for specific materials, systems and methods

We require information from authors about some types of materials, experimental systems and methods used in many studies. Here, indicate whether each material, system or method listed is relevant to your study. If you are not sure if a list item applies to your research, read the appropriate section before selecting a response.

Materials & experimental systems

n/a	Involved in the study	
☐	☒	Antibodies
☐	☐	Eukaryotic cell lines
☐	☐	Palaeontology
☐	☐	Animals and other organisms
☐	☐	Human research participants
☐	☐	Clinical data

Methods

n/a	Involved in the study	
☐	☐	ChIP-seq
☐	☒	Flow cytometry
☐	☐	MRI-based neuroimaging

Antibodies

Antibodies used	Anti-human/mouse CD49f-eFluor450 (Ebioscience, San Diego, USA, clone GoH3) 1/50. Anti-Integrin-alpha 6 (avian, P2C62C4, DSHB), AntiPou3F2 (PCRP-POU3F2-1A3, DSHB) 1/50, anti-histone h3 phospho S10 (Abcam ab47297) 1/100. anti-mouse HRP (Abcam Goat Anti-Mouse IgG H&L HRP ab6789) 1/500, HRP-conjugated anti-digoxigenin antibody (Sigma-Aldrich 11207733910 Roche) 1/500, HRP-conjugated anti-fluorescein antibody (Sigma-Aldrich 11426346910 Roche) 1/500 or unconjugated anti-DNP antibody (Vector labs SP-0603-1) 1/100, anti-rabbit HRP (Goat Anti-Rabbit IgG H&L (HRP) (ab6721) Abcam) 1/500
Validation	Anti CD49f eFluor 450 was validated by isotype-control staining (Figure S1C) and enrichment for IA6+ cells was confirmed by qPCR (Figure S1D). Antibodies used in Immunofluorescence were validated by secondary-only controls. AntiPou3F2 (PCRP-POU3F2-1A3, DSHB) was validated: "This antibody has been characterized by the NIH Protein Capture Reagents Program RRID: AB_2618965" dshb.biology.uiowa.edu/PCRP-POU3F2-1A3. Anti-Integrin-alpha 6 (avian, P2C62C4, DSHB) was validated "Stains chicken embryo cryosections" https://dshb.biology.uiowa.edu/P2C62C4. Antibodies used in FISH were validated by using sense probes and slides without probes. Anti-digoxigenin: "The polyclonal antibody from sheep is specific to digoxigenin and digoxin and shows no cross-reactivity with other steroids, such as human estrogens and androgens."Anti Fluorescein: "The polyclonal antibody reacts with free and bound fluorescein." https://www.sigmaaldrich.com/catalog/product/roche/11426346910?lang=en&region=US. For other commercial antibodies, validation information can be found at: Anti DNP https://vectorlabs.com/anti-dnp-dinitrophenyl-unconjugated.html#documents, anti-rabbit HRP (Goat Anti-Rabbit IgG H&L (HRP) (ab6721) Abcam) https://www.abcam.com/goat-rabbit-igg-hl-hrp-ab6721.html.This product was prepared from monospecific antiserum by immunoaffinity chromatography using Rabbit IgG coupled to agarose beads. Anti-histone h3 phospho S10 https://www.abcam.com/histone-h3-phospho-s10-antibody-ab47297.html. Goat anti Mouse HRP https://www.abcam.com/goat-mouse-igg-hl-hrp-ab6789.html. This product was prepared from monospecific antiserum by immunoaffinity chromatography using mouse IgG coupled to agarose beads followed by solid phase adsorption(s) to remove any unwanted reactivities.

Eukaryotic cell lines

Policy information about cell lines

Cell line source(s)	*State the source of each cell line used.*

Authentication	*Describe the authentication procedures for each cell line used OR declare that none of the cell lines used were authenticated.*
Mycoplasma contamination	*Confirm that all cell lines tested negative for mycoplasma contamination OR describe the results of the testing for mycoplasma contamination OR declare that the cell lines were not tested for mycoplasma contamination.*
Commonly misidentified lines (See ICLAC register)	*Name any commonly misidentified cell lines used in the study and provide a rationale for their use.*

Palaeontology

Specimen provenance	*Provide provenance information for specimens and describe permits that were obtained for the work (including the name of the issuing authority, the date of issue, and any identifying information).*
Specimen deposition	*Indicate where the specimens have been deposited to permit free access by other researchers.*
Dating methods	*If new dates are provided, describe how they were obtained (e.g. collection, storage, sample pretreatment and measurement), where they were obtained (i.e. lab name), the calibration program and the protocol for quality assurance OR state that no new dates are provided.*

☐ Tick this box to confirm that the raw and calibrated dates are available in the paper or in Supplementary Information.

Animals and other organisms

Policy information about studies involving animals; ARRIVE guidelines recommended for reporting animal research

Laboratory animals	this study did not involve laboratory animal strains.
Wild animals	Botrylloides diegensis colonies were collected in Santa Barbara, CA and maintained in flowing seawater at 19-23C. Colonies were glued onto glass slides. Animals were fed daily with live algae.Growing colonies were subcloned onto individual slides to generate sets of genetically identical colonies. Only healthy, growing colonies were used for experiments. Colonies were maintained in flowing seawater until they died of natural causes (4-9 months).
Field-collected samples	Botrylloides diegensis colonies were collected in Santa Barbara, CA and maintained in flowing seawater in the dark at 19-23C. Colonies were glued onto glass slides. Animals were fed daily with live algae. Growing colonies were subcloned onto individual slides to generate sets of genetically identical colonies. Only healthy, growing colonies were used for experiments. Colonies were maintained in flowing seawater until they died of natural causes (4-9 months).
Ethics oversight	For studies on invertebrates, no ethical approval is needed.

Note that full information on the approval of the study protocol must also be provided in the manuscript.

Human research participants

Policy information about studies involving human research participants

Population characteristics	*Describe the covariate-relevant population characteristics of the human research participants (e.g. age, gender, genotypic information, past and current diagnosis and treatment categories). If you filled out the behavioural & social sciences study design questions and have nothing to add here, write "See above."*
Recruitment	*Describe how participants were recruited. Outline any potential self-selection bias or other biases that may be present and how these are likely to impact results.*
Ethics oversight	*Identify the organization(s) that approved the study protocol.*

Note that full information on the approval of the study protocol must also be provided in the manuscript.

Clinical data

Policy information about clinical studies

All manuscripts should comply with the ICMJEguidelines for publication of clinical research and a completedCONSORT checklist must be included with all submissions.

Clinical trial registration	*Provide the trial registration number from ClinicalTrials.gov or an equivalent agency.*
Study protocol	*Note where the full trial protocol can be accessed OR if not available, explain why.*
Data collection	*Describe the settings and locales of data collection, noting the time periods of recruitment and data collection.*
Outcomes	*Describe how you pre-defined primary and secondary outcome measures and how you assessed these measures.*

ChIP-seq

Data deposition

☐ Confirm that both raw and final processed data have been deposited in a public database such as GEO.

☐ Confirm that you have deposited or provided access to graph files (e.g. BED files) for the called peaks.

Data access links<br>*May remain private before publication.*	*For "Initial submission" or "Revised version" documents, provide reviewer access links. For your "Final submission" document, provide a link to the deposited data.*
Files in database submission	*Provide a list of all files available in the database submission.*
Genome browser session<br>(e.g. UCSC)	*Provide a link to an anonymized genome browser session for "Initial submission" and "Revised version" documents only, to enable peer review. Write "no longer applicable" for "Final submission" documents.*

Methodology

Replicates	*Describe the experimental replicates, specifying number, type and replicate agreement.*
Sequencing depth	*Describe the sequencing depth for each experiment, providing the total number of reads, uniquely mapped reads, length of reads and whether they were paired- or single-end.*
Antibodies	*Describe the antibodies used for the ChIP-seq experiments; as applicable, provide supplier name, catalog number, clone name, and lot number.*
Peak calling parameters	*Specify the command line program and parameters used for read mapping and peak calling, including the ChIP, control and index files used.*
Data quality	*Describe the methods used to ensure data quality in full detail, including how many peaks are at FDR 5% and above 5-fold enrichment.*
Software	*Describe the software used to collect and analyze the ChIP-seq data. For custom code that has been deposited into a community repository, provide accession details.*

Flow Cytometry

Plots

Confirm that:

☒ The axis labels state the marker and fluorochrome used (e.g. CD4-FITC).

☒ The axis scales are clearly visible. Include numbers along axes only for bottom left plot of group (a 'group' is an analysis of identical markers).

☒ All plots are contour plots with outliers or pseudocolor plots.

☒ A numerical value for number of cells or percentage (with statistics) is provided.

Methodology

Sample preparation	Samples were prepared as described in the Methods section. Blood was pooled from genetically identical, stage matched samples and diluted with filtered seawater. Anti-CD49f was diluted at 1/50.
Instrument	BD FACS Aria III
Software	BD FACS Diva
Cell population abundance	IA6+ cells comprise 9.7% of total blood cells (Figure S1C).
Gating strategy	A live cell gate was selected based on forward/side scatter, and samples were gated as IA6 (CD49f) + based on isotype control staining.

☒ Tick this box to confirm that a figure exemplifying the gating strategy is provided in the Supplementary Information.

Magnetic resonance imaging

Experimental design

Design type	*Indicate task or resting state; event-related or block design.*

Design specifications	*Specify the number of blocks, trials or experimental units per session and/or subject, and specify the length of each trial or block (if trials are blocked) and interval between trials.*
Behavioral performance measures	*State number and/or type of variables recorded (e.g. correct button press, response time) and what statistics were used to establish that the subjects were performing the task as expected (e.g. mean, range, and/or standard deviation across subjects).*

Acquisition

Imaging type(s)	*Specify: functional, structural, diffusion, perfusion.*
Field strength	*Specify in Tesla*
Sequence & imaging parameters	*Specify the pulse sequence type (gradient echo, spin echo, etc.), imaging type (EPI, spiral, etc.), field of view, matrix size, slice thickness, orientation and TE/TR/flip angle.*
Area of acquisition	*State whether a whole brain scan was used OR define the area of acquisition, describing how the region was determined.*

Diffusion MRI ☐ Used ☐ Not used

Preprocessing

Preprocessing software	*Provide detail on software version and revision number and on specific parameters (model/functions, brain extraction, segmentation, smoothing kernel size, etc.).*
Normalization	*If data were normalized/standardized, describe the approach(es): specify linear or non-linear and define image types used for transformation OR indicate that data were not normalized and explain rationale for lack of normalization.*
Normalization template	*Describe the template used for normalization/transformation, specifying subject space or group standardized space (e.g. original Talairach, MNI305, ICBM152) OR indicate that the data were not normalized.*
Noise and artifact removal	*Describe your procedure(s) for artifact and structured noise removal, specifying motion parameters, tissue signals and physiological signals (heart rate, respiration).*
Volume censoring	*Define your software and/or method and criteria for volume censoring, and state the extent of such censoring.*

Statistical modeling & inference

Model type and settings	*Specify type (mass univariate, multivariate, RSA, predictive, etc.) and describe essential details of the model at the first and second levels (e.g. fixed, random or mixed effects; drift or auto-correlation).*
Effect(s) tested	*Define precise effect in terms of the task or stimulus conditions instead of psychological concepts and indicate whether ANOVA or factorial designs were used.*

Specify type of analysis: ☐ Whole brain ☐ ROI-based ☐ Both

Statistic type for inference (See Eklund et al. 2016)	*Specify voxel-wise or cluster-wise and report all relevant parameters for cluster-wise methods.*
Correction	*Describe the type of correction and how it is obtained for multiple comparisons (e.g. FWE, FDR, permutation or Monte Carlo).*

Models & analysis

n/a	Involved in the study
☐	☐ Functional and/or effective connectivity
☐	☐ Graph analysis
☐	☐ Multivariate modeling or predictive analysis

Functional and/or effective connectivity	*Report the measures of dependence used and the model details (e.g. Pearson correlation, partial correlation, mutual information).*
Graph analysis	*Report the dependent variable and connectivity measure, specifying weighted graph or binarized graph, subject- or group-level, and the global and/or node summaries used (e.g. clustering coefficient, efficiency, etc.).*
Multivariate modeling and predictive analysis	*Specify independent variables, features extraction and dimension reduction, model, training and evaluation metrics.*
