# Supplementary material for: Integrin-alpha-6+ Candidate stem cells are responsible for whole body regeneration in the invertebrate chordate Botrylloides diegensis
Source: Nat Commun. 2020 Sep 7;11:4435. doi: 10.1038/s41467-020-18288-w (PMC7477574; doi:10.1038/s41467-020-18288-w)
Supplement: Supplementary file 4 — Description of Additional Supplementary Files [file 41467_2020_18288_MOESM4_ESM.pdf]

## Description of Additional Supplementary Files

**Supplementary Data 1. *B diegensis pou3* mRNA sequence**

**Supplementary Data 2. *B diegensis integrin alpha 6* mRNA sequence**

**Supplementary Data 3. *B diegensis piwi1* mRNA sequence**

**Supplementary Data 4. *B diegensis piwi2* mRNA sequence**

**Supplementary Data 5. Primer and probe sequences**

**Supplementary Data 6. pou3 protein sequences**
