# Supplementary material for: Integrin-alpha-6+ Candidate stem cells are responsible for whole body regeneration in the invertebrate chordate Botrylloides diegensis
Source: Nat Commun. 2020 Sep 7;11:4435. doi: 10.1038/s41467-020-18288-w (PMC7477574; doi:10.1038/s41467-020-18288-w)
Supplement: Supplementary file 5 — Supplementary Data 1 [file 41467_2020_18288_MOESM5_ESM.rtf]

Supplementary Data 1. B diegensis pou3 mRNA


B. diegensis POU3 partial CDS confirmed by Sanger sequencing:
gaaataacttttacgcccatgtgtcattgaccaatgaaaatggaagaagtgtgatcgcac aaggaaatgaatgcagcggcaagcaatctcccgaatacaaagaatatgaaacgccaatta aagaattgacgtacatgagtctgcatcctagaacggtcacaagtaatggatactattcac agtcgtcaacattcggtgaaaatttcagacatttcccaactcaatctccatatacaggcc atgatcataacccagggtacaattacaacagctacccacctacgttgatacccgctgatt gtttacaaggatccttacaatcaagacattcgtttaactcgacgtttgcacctgagtcat acgcatCtgagcaaaactccgatcatccacgaatttcaaggtctgcgcccccaaacgtta cgattactgaatgctctgatacacactacaatgcaaattcagtgaaatgtttCtcagacc ggcacagcgaatattcgtatccacatcttccatctccagcaaacatgggcgttgtatgca agcgagaaataacaactccatccccacaactgcgacatgaaataagcagttcatggaggg gacaagagttgtgtccaccatcgtacttgcaccaagacacaccacagtatcgatattctt atcaagcaaactattggccactatctcctgcaaactcaacatcttgttcatatcaaaagg cctcttcaaatcggtttgtgaagcaagagaggtttcaagattaccaaacgaatacaccga tgcaaaagttgccgtttcgaaacttctgcacggcggcagaacgcggattttcaaatgaaa cgcaatttgaacaaaagtttacatccgacgaatctatgataggcacagaatcgtctgacg acatgcgaattttcgcaaacgttttcaaagctcgtagaataaaactcggtttcactcaac atgacgttgggttagatttaaaaaagtttcaagggtccgcattcagtcaaactacaatct gtcgattcgaggctgggggtttaagcatcaaaaacatgaataggttaaaacctctattaa caatgtggctacgacacaacgataccgagcatatttcaaccttgagggatactgatcgat cgccattagacaacgcaaccactcgaaaaagaaagaaacgaacgtgtatagaaccccaaa ctaaacttgctcttgaagaaaaatttaggaatgatcagaaaccaacaacagttcaaattg ctaaaattgcagaagaactgtcattagataaagaggtggtgcgaatttggttttgtaaca ggaggcagaaagagaaaaaggcaacagttgaaattgtgcagagggatgtggcataa
