# Supplementary material for: Integrin-alpha-6+ Candidate stem cells are responsible for whole body regeneration in the invertebrate chordate Botrylloides diegensis
Source: Nat Commun. 2020 Sep 7;11:4435. doi: 10.1038/s41467-020-18288-w (PMC7477574; doi:10.1038/s41467-020-18288-w)
Supplement: Supplementary file 6 — Supplementary Data 2 [file 41467_2020_18288_MOESM6_ESM.rtf]

Supplementary Data 2. B diegensis integrin alpha 6 mRNA

[organism=Botrylloides diegensis] integrin-alpha-6 mRNA, partial cds
GTGGGTTTAGGTTTACTTTTCGCAAAAGGCAAAACGCTCTGGAGGTGGGAAGAATGACTTCAACCACTGAGCTGGAAAAGCCTCCGATCGTTGCCAGTAGCGTTTTTCCGTTTATAGATTATCGTGATATGCAGTGACCGTCTTCGGTGTTGGGCTGGGTTTGGGTAGCGGCCGATGACTGTTCTCATTAGTGTTGGCGATCCAGTCGGTGTTTTTGTCGATGTTCCAGCCGTTGAGTGAGACATGGCGTGCTTTAACCCGTCCACATTCAAGTGCAACAATAATATAGTAATTGTCAGCACAACGTTACTGCTGCTTTGTCTTCATGTCGTCAGCGTATGGGGATTCAACATCGACGAAAGGCATCCAATCGTGAAAACTGGACCCCCTGGCAGCCTTTTTGGATTATCTGTCGCTGAACACCACGTCAATGAACGTATTCAAAGCAAAGATAATGCCTTACTCATTGTTGGTGCTCCGACAGGCGTAGCTTATGGTAGCTCAGACAACACTCAATCTGGCGCTGTTTATAAGTGTTCTGTTTTTTCTGATGCCAATTGTACTCTTGTTCCAATAATACCTCCAAATAGTGGACGTGTTGAAAATACCACAAGTCAGTGGTTAGGGGTATCTGTGAAGTCACACAAGCCTGGTGGTCAGATTTTGACTTGTGCTCATCGTTACACACTCGTTGGGTCAGACTGGGTAGCGCCCGTTGGACGATGTTTCATGCTTGAGAAGGACACGACTCCGGTACAAGATGAATTTTCAGCTGAATATGTACCGTGCGAAGATAAGCAAGATGGTCTTGGCCGCTATAGCCATGAGGGATATGGATACTGCCAGGCTGGAGCCTCGGTGAATTTTGCTGATGTTCCTGCTAGCGATGATGGAGAATCGTATGTCTTGATTGGAGCCCCGGGGAGTATCCACTGGAGTGGCGCTGTTCTTGCTACAAGAAAGGGAGGTGATTTTGGTCTGTCGGTTGAGAAAGTATGGTCTGATAAGGATCTCACCATGACAAATTATCAAATGGGCTCTTCTGTTCTGGCTGGTTTTATCTACAGAGAAGATGCTGTAAATTTTGTCACTGGAGCACCCGGCGCTAACACTACAGGTGCTGTGTATATACTTGAAAAAACGACACCCGACGTTACTAATTCTGATGGAGATAGTTACCTGAGAATTGTCGAGACGGTAAATGGTGATAAGGTGGCTTCCAGATTTGGACACGACATACTTTTGTTAGATGTTACAGGAGATGGGAAACTTGACCTGATCATCGGTGCACCTCAGTTTTATGATAGAAATGATCAAGTTGGTGGGGCTGTCTATGTTTACGTGAATAAAGGTTTGTCAACCATTGGTCCATCACCTACTCAGAGGTTGTTTGGTAACATTGACTCCTACTTTGGGATGGCTGTAGCAAGTGCTGGAGATGTTAATATGGATGGAGTTAATGACATTGCAATCGGTGCTCCGGGTGGAAATAAATGGACTGGTGTTGTGTATATCTATCATGGGGATAGTAGTGCTGACGGCATGGGTGTTGGTGCAAAGCCGTCACAGATCATAGAAGCAAGTAAAATATCAGCACTGGGAGGAAATAGTTACGGATTGGGATATTCCTTGAGCGGTGGGCTTGACATGGACTTGAATGGTTATCCCGACATCGCTGTAGGAAGTTTGTCAGACTCTGCTGTTGTATTTTTCTCCCGACCTGTGGTCAATGTTACTGGTACGATAACCGGTCCAAACAAGAAGATCGAGCTGTCGGAGGATCCATCAGAACAAGTACTTGATATCGAAATCTGTCTTCGTTACACTGCCTTACCGCCCAGCTTTGATGAAAGAGTACGCGTAGAGGGATATGTGGAACTAGATTCTGGAAGGGTTGAACGGGGATTACTATCAAGACTCTCATTTAAACGCTCTTCTACTAAGGAAAGCGAAGGCCAATCGAAAAAACCATTTGTCATGACTCTTCATCGTCAATCAGCTCAAAGGGACAAATGCGACACGTATAAAACATACATGAAGAATGACATCAGAGATAAGCTGACTCCTATTGATTTGAAATTGACATTTACTACTCCCAATGACCCGCCAAAAAAGAGGCGTCGACGCCGTGATACTACTTATACACCACCTATTCCTATCATGAATACTGCTATCACGGACACGGCTGTTGCGGAGATTGAATTTGCCAAACGGTGTGGGGATGATGAAATATGCAGTAGTAACTTGGAAAAGAAGGCTTATTATCAAGTTTTGAAGAATGGAAACTGGTCAGATTTGACAAGGAAGCGCAATGGACAGCCTTTGTTAATTTTGGGAACGGAAGAACAAATTGGATTGGTTATAGAAGTGACCAACCAAAATGGCGAGGACGCACATCAAGCACGTATGAACATTGTTCTTCCTGATGAAGTGGAATATCGTAGGATAGAGTTGATGTCGGGAAATGCTATCCATTGTGATCCAGACACCAAGAATCGTTCACTTGTAGTTTGTCATCTCGGCAACCCATTTAAAGAAGAAAGCAGTGTTAAATTCAAACTGAAGATGTCAAAGACACAAAGCATTCAGAAGGCGACACAATTTGCTGTGAAACTTCAACTTTTAACGACAAGTCAACAG
AAAATCCCGGATCCTGGTACAGAGTACTTGGTCTATGTGGAAGTGGAAGCACAACTTGAACTTGTTGGCTATCCAGATAGGGAGCAAGTGGATTTCTCAGGAGTGGTGATCGGAGAATCAGCCGTAATGAAACCGACTGATGCAGGAACATTGCACATGCACAAGTATGAGATAAAGAACTCTGGAACAGGAATTGTTGAGGATGTTGCGTTGAAAATAATGTGGCCACAGGCTATCATAAACGGCAAATGGCTCTTTTATCTTCTCAAAGCAGAAGTTCAAGGTTTTGGAAATTGCTCCTCCCCTGGTAATGTGGATCCGTTGCAATTAAACCTCAAAAGCAAACGTGCAAAACGGGACGTGGATCCACCTCCTGGAAATCTTAAAGATGGTGGAAGCCCCATTATTAAATACGCATCGCTTAATTGTCGTGATGGAGGTGGACTAAATTGTGTTGAAGTGGTCTGTTTCCTTGGAGATCTTGCATCCAAAGTGACAGCTCGGGTCGACCTAACAGCAATTTTATGGAATAGCACGTTTTTAGAGGAATTCAGTAATGTAGCTGGGGTTTACGTTGAATCGGATGCCAAGGTCAGCATCCTGCAAGAAAATATCAAATTTTCTGAAGAATCCACGTTGGAAAGATCAACAACCACCACTGTCTTGCCTCTCGAAATATTTGTGCCTAAACAACCCATTGAATGGTGGATTATAGCAGTAGCGGGTGCAGCTGGACTCATCCTTTTGGTTTTCTTGGTTTTGATTATGTGGAAGTGTGGCTTCTTCAAACGCCAGACGCTTGTCGACTATCAAAAAGCACGCAGACACAAGCAGGCTTCCAAGAAGGCTGAAGAAAAGGAGAATTTGTATTGAGTATGGTGTTATGATTGAAAGCGATCAATCTCTACATAGTGAAGGTAAACACGGATCAAACGATCAGTGTTGGCGTTGTAATCGTCGTGACAGTTGTTGCTGTGTGCTGAGCGGAGACCTTTTCTGTAATAATTCGTTATTTGCAAATTGATGGAATGTATTTATCCGGCACATGTTGTTATCGTGATCTTTTACTTTGCTGCGGTGCTACTACGACTCATACGGCTCATTTTTTATGCATTTTTTATGACCCTGTTGCTTTTTTAAATTTTACTGCCTTCATATATGATAATCTATCTAATCAGATTAACATTGCTCTTATACTAAGTTGAATTGATTTTAATAGTGACATGGACTCACCTATTGCATTTGAGAAAATTTTGGAGACTTTTTGTCAACTGTTCAAGTTTTCATATCACCATCGTATCATGTATAACGGTCGGTTCCAGGGATGGGTCACGGTGAAAATTGGCGCCGCGAATACCCGTGAATATCACCGATTCACGAATTAAGATATTACATTTGCCTCTGGTATTCGGAGCTGTTAGATTTTGAATTTCTGACATGGTAACTTGACATGCCTTCAAGGTCTAGCTTCAAATGGTGTAAGTTTTTTTTTGCCCCTGTTCACCATGTTATCTGAATTCACCGAATGAGCGCTGCAGTTGAATGAATGCAGATGACCACACCCCTTCAAGAATGCCTGTCGTTGTCGCTGATTTTTTGACCGCTACTGATTGTCAGCAACGACCTATGATCACAAATATTGTTCTGAAAAGTAATAACGAATCAGAGAAAATCCCAACTATCCCTGATCGGGAAATGCACTGCTCACAATACGCGAGGTTGTTCCTGCGGTGAAACGGCAAATTTTCCAACTAAATGTGGACGACTAAAAAAATGTCCTTTCTCCTAATCATTGCCGTTTAACATCTTCAGTCACTAGCAATGGAATTTAGTTACACGTGAAATTTTGCGGCACGTAGGGCTCGTGCCGTGCTTGCCTTCCGCTTGGTAGGTTGTTCAAGTTAATAACTAGCGAATCATTCACCGTACACGCCCAGGCATCCAGGTTTCGCTGAATATTTGAACAAATCCAGTATGTTTGGCCAACCCTCCAGTTGTGTTGAGAACCCGATCCGTTGACTTCATCTTTGGCGACTTGAAATATTCCGTTTCCTAGTATGACACCCTTGCAGGCCAGGTTGTCGCGTTTGGCCATTTAGGTTGTCACAGTTCATACCACTACTCTAACCGGTACACTAGCAAAGTTTATGAGATCGTGAACATATGATGCGTTTTCTTAGCAATTCATGATATTCTATTTCCTTGGGCGACATAATGAAGGAAAAGGTGCTTCCACCAAATTCATATACAGTAATAAATGATAGTTCATATCACAAGTCCTCATT
