# Supplementary material for: Integrin-alpha-6+ Candidate stem cells are responsible for whole body regeneration in the invertebrate chordate Botrylloides diegensis
Source: Nat Commun. 2020 Sep 7;11:4435. doi: 10.1038/s41467-020-18288-w (PMC7477574; doi:10.1038/s41467-020-18288-w)
Supplement: Supplementary file 7 — Supplementary Data 3 [file 41467_2020_18288_MOESM7_ESM.rtf]

Supplementary Data 3. B diegensis piwi1 mRNA sequence

 [organism=Botrylloides diegensis] piwi1 mRNA, partial cds
GATCTGTTGTATTAAGGTTGCTGCAGAAGTATATGTAAAGTAAGCAGTTGTTTTATGGTTGAATAATGGCTGAGCAAAGGGCGAACCCAGGAAGAGCGAGAGGAAGATCTCGTGGACGAGGCCAACCCCTAGTACCTGATGCCTCTGCTCAACAACCTGCTGTGGGGAGAAGAGGAGGATCAGATGCAGAGGTTGGAAGTAGCATGATTGGCTCAGGCAGGGGAATGCAAAGGGGTTTGCATCATTCCACACAAGGACCAAGGGGTGATGCCGGCGTCCGAGAAATTACTAGAGGAGTTGGCCGCATGACTTATTCTGGTAACCGTCAAGTCATGCTTCTTGGAGCAAGGAGAATGTTGGACCCATCCATTTTTGATACTGGCACAAGGCCAGATCACATCAAGGACAAAAGAGGAAAGGATGGAAAGCCTGTTCAGATCTTGACTAATTACTTTCCACTCGTGTCATCCAAAGCATGGCGTTTATATCAATACAGGGTGGACTATGAGCCAGATGTCGACCATAAAGGGGCACGCCGGGGAATGCTCAGGGATCATCTAGATCTGATTGGCAATACTTATATGTTTGATGGATCAACTTTGTACACATTGAGAAGACTGCCAGAAGAGGTCAACATCGTTCACTCCAAAAGACTTTCCGACAATGCAACGATCCAATTGAAAATCGCCAAGACTGCTGAGCTACCGCCAAACTCGCCATTGAGCATTCAGCTTTACAATTTGATATTTCGCAGTGTATTGAAGAAGCTTGGGTTGGAGCAGGTCGGCCGACACTACTATGATAGCCAGGCCAAGTTTACCGTCAACTGTGACAATATGAGATTTGAGCTGTGGCCTGGTGTAATCACAAGTATTCTACAGTATGAGAAAGAGAGCGTCATGATGTGCACTGAAATTTCCCATAAGATGATGCGGGTAGAAAGTGTTCTGACCATTATGAAACAAAAATATGAGCAGTCGAGGCAGAGACGAACCAACTTCCATCAGGAATGCCAGCAGTTCTTGCTCGGACAAATTGTACTAACACGGTACAATCACAAAACATATCGTGTCGATGGCATCGAGTGGAAGATGAATGTCTCAATGAAGTTCAAAAAGGGAGACGAGGAGATTTCCTATGTTGACTATTATAAAACGCAATACAACATTACGATTAAAGAGATGGATCAACCTCTGTTGTTGTCTCGTCCAAAGAAAAAGGAAGCTAGAGATGGACTGGAAGCGATTCACTTGGTGCCCGAATTATGTACTGTGACTGGACTATCAGATGAGCAACGGGCCAATTTCTCTGTGATGAAAGCACTCGCTGAACATACCAAACAGGGACCTGCCAAACGTGTGAACGCTCTGAAGAATTTTATTCAGAGAGTGTGCGGACACCAGGAAGCGACTGACTTGCTTAACAAATGGGGGCTTCATTTTGAAAGAGATTTGGTAGGAGCACCAGGTCGCGTTCTACCTCCAGAGCAGCTCATGTTTGGAGAGCGTAAGTGCATCAATGGAGGTCAGTATGCTTCTTGGGACAACGAGCTCCGCAACTGCAAGCTACTGAAATGTGTGGACCTCCGAAATTGGCATCTCATCTGTCGCAGACAGGACCAGCAGATGGCGAACGGTCTGATTCAAAAGATGTGCCAAGTTTCAAAAAATATGGGTTTTGATATGTCGCGCCCTGTTTTGACTTGCATCGAACAGGACAGGACTGATGTGATTCTGAGCACCATTAAAGACATATGTGCCAAGTCTAGCAACTGTCAATTGATACTTTGCCTGCTGTCCAGTGATCGAAAGGAACGCTATGATGCTATCAAGAAAGTGTGCTGTGTGGATGTACCGATTCCCACTCAGGTGGTCAAGACCAAAACAATCTCGAAACCTCAGAGACTGATGAGTATTGCAACAAAGATTGCTATACAAATCAACTGTAAGCTTGGAGGGGAAGCGTGGGCTGTGAATATCCCGCTTGGTGGTACAATGGTCATTGGAATCGACACTTACCATGACTCTATCCATAAAAGTCAGAGCGTCGGTGGGTTTGTTGCCAGCATCAATCGAGGCTTCACCAGATGGTACTCATCCACTACAACCCAGCAAGCAGGTGTTGAACTCATTGACGGATTAAAAGTCTGCATGGTCGGGGCCCTGAAAAAATACAGAAGTGAGAATGGTGAATATCCGAAAAAAGTTTTCGTATTCCGCGATGGTGTTGGTGATGGCCAGTTGTCCATGGTGAGAGAACATGAAGTGCCTCAAGTCATCCAATGCTTGAGAGACCCGTCCAATCCGAATGCCAAACCTATTCCACTGTCTTATATTGTGGTGAAGAAGCGCATTAACACGAGACTTTTCACAAGTGGTAGACAAGGCATGGCAAACCCACCTCCTGGCACCATTGTTGATGATGTTATAACTCGTCCGGAATCGTATGATTTCTTTGTGGTGAGCCAGAATGTGCGGGAAGGGACTGTGTCGCCTACACACTACAATGTAATATTTGATGAGTCCGGATTGGCTCCAAATCACATGCAACGCTTGGCATACAAACTCTGTCATGTCTACTACAATTGGCCGGGCACAGTGAGAGTTCCTGCTCCATGTCTGTATGCTCACAAGTTGGCTTTCCTTGTTGGTCAAAGCGTCCATCGAAATCCTGCAGCATCGCTGGCTGACAAATTGTATTTCTTGTAATCATGTATCAAGTGTGCTTTTCTCATAACGGTGTGCCTTCGTTGATAGTTCCAGTTTTTATCATTTTTGACACAAGGTGATTTGATCATGTATAGTGATAGCAGCTATACAACATTCTGTTATTTCTTGAGGTTTTGAACTGTTCCTGTTTGTGCACTGCCTTTTTCTTGAGCTGGGCGCGACCAGCCTGTAATTCGCCGATTGTGGGCGATGCCGTGTTGTGGTGATGATTTCTTCGCTTGAAACTGGCCGCTTGGTCAACTAGGGGTGCGTTTTATATTCATAAATGTGAAATTGATAATTGGATTGGATCTGTTACGAGCTAACGATAAACTGCCCGATTCATGCTGAACTGTTACATTCGCCAGTATGTGGTGATGATTTCTTCGCTTGAAACTGGCCGCTTGGTCAACTAGGGGTGCGTTTTATATTCATAAATGTGAAATTGATAATTGGATTGGATCTGTTACGAGCTGACGATAACTGCCCCTGATTCAGGCCGAACTGTTACATTCGCCAGTATGTGTGTAATCGTTTTATGTAACTTGGCGATTGTAACGTTTTATGATATGCTGTGTTGGTTGGATTTCGTTGGTGAAATGAAGCTTGTCTGTCATGATTGCGTTGTTGCTAGCTGGTCTTATTTGATGACAAGCGGGTGCACCTATTTTAGTTGGTGTTTGGAGTTTACGGGACTTCGGAAAAATTTGTCCCTGATCACGAGGTTGATACTTTACCTCGTGAATTATTGCAATGCGATACGAATCCATTCTAGAACCGCCGCTGGTTGCGGATGGTCACGGATTCCCTTTCAGTTCATAATAATTTTTTGTTCAAGTAGGCCTATCGATTCAGCGTCGACTTATTAGCATGAAGCTAGTAGCATTAGCATGCCACAACAATCGTAGTCCACGATTTAGTCGGCGAAACGTGTCCATCCATAATCCTAATGCAATAACGATCTGTCATCAGGTGCACAAGCCCCATTTCAAGGATCCGGATATCAAATCCGGGCGGCCCTTGAGAGAGGTGGCCTGCTGAACATTGGATTGAGTTGCTTTATTAGAAATGTAAAAGCCTACACTACGTACGACCTAGACCCTGGCGTTCGCTTCTCACGGGAAGTGAGAAAACCCGCCCCGAACTAGCTTCCGTTTCTCATTCACTCTGGTTTTCGATATGGGTATTAGATGCTAGGCCTATTACTATTGGTCTGCAAACAGCCTACATCTAGACTAGCTCTGTCGCTTGCTTTGCCAGTTTTAATGCAACAAGCTCGATTTCTTCTTTGTAAATGTTAGGACTGGTTCAGTGGTGCGAATTCACTCTTTTGGCTATTTTCAGGGCATTCTGTGGAACCTAAACTAAGCTCTGTTGCTACTATAAGCTAGGCTAGGACCGTGTATAGTCAGCTAGAGAGGGC
