# Supplementary material for: Integrin-alpha-6+ Candidate stem cells are responsible for whole body regeneration in the invertebrate chordate Botrylloides diegensis
Source: Nat Commun. 2020 Sep 7;11:4435. doi: 10.1038/s41467-020-18288-w (PMC7477574; doi:10.1038/s41467-020-18288-w)
Supplement: Supplementary file 8 — Supplementary Data 4 [file 41467_2020_18288_MOESM8_ESM.rtf]

Supplementary Data 4. B diegensis piwi2 mRNA sequence
B. diegensis piwi 2:
CCGAGATTACTGCAATCTCTGCCGTTGCAGGGTCCCTTGACACTCCGTCGATGTTTTCTCTGAATATAAGCCAAATTCTC
TCTCAATAAAAAAATGTATCGCGAATTTTTTTGATTTTACAATCTCTTCGCTGCTGCCTTATTGTTTCATTTTGCATGGC
TTCCTCTGTCCCAGCCAATGTCGGTCGATAAAGCAGACCTATCTCATACAGGTACCGCGCCTACAGTTCAACACCACACG
CCTAATCAGAGAAAACCCCTATGTTATATGACCCAATGATATATATGACAGATTAGAGAGTCTGTAGGCCTACTGCCAGA
CCAGTGGCCGATGTTAGGCCACCTTTTCGAACTAGAACGGCGCAACTCCGTTGCCTATCCATGCGGAAAGGACTGAAGCA
CTGAGCCGAATCGTCCGGATCTTTGACACGGGGCTCTACACAAGATGGCGTGCGCGTACCGTAGACAAAGACCTAACTGT
TCCAGGTCCCACGTGATGGACCCACCTGGTAGTGAACCCTGAACCGTTTGTTGGTTTGTTGACTTGTTGAGCACAACGGA
GAAAAGGAAGTGGTACAGTAACTGGAAGGTGAATTAGTGAATATTTCGTTTATTATCGCCATTTTGTGCTTTGAGGCGGA
GTTTATCCCGCGGACTATCCATCGCATCCGCATCCCGTTGAGGGCAAGCCCTCATTTCCTTGATGCGAATGCATAGCTGT
TTTTCTATGTTGCTAGTAGCCTACGGGTGGAAGTTGTTGGTCGTTTGCAATACTGCTTGCCCATATGTAAAAGTAGTAGG
CTATGATTAAAATGCCACCCACTACCAAAACATCTAAGCTGGCTGAGAATTATGCAATCTAGTCTACCCGGTGTGCTAGT
CATGTTCGCCGAGAATCCGACCACCGTTTTGATGGGATAGATGCACTTGATGCAAAAACTACCTCAAGTCAGTCCACGCA
ATGTACGGATACATTTTGAGATCAATGTTTCAATTGAGTATCCGCACCCTAACACGGTCTAGCCTTGGCTTGTAGAGTTT
ACCAAGTTACAAAAACGTCGCCTTTGTCGGCCTGGAGCCCAAAAATGTAGCAATAGGCTACAAAATGTACTGCTTATCCT
TGTGTTGAGAGCCTTGAAAGTACTGAAATGCGGAGGATAAGTTATGACGTCATATTGATACATTTTCAAGTGTTACCAAC
AATGATATCTAGCATCTGCGCACCAATTTTGAGACCAATCAGGTCGGCCCGGTAGAACTCAAGTTATAACATAGAAAATC
ATCTTCCAACTACATATTATAGATTTGCTGTGTTATGAGTATGAATCTCCACGCTCTGGCAATTGTGGCAGCGCCTGGCC
GCCTGGCTGGGAAGCTACAGTCATCATATTTAACGCACTTAGTAGCATGGCAGCAAGTGCCTCAACCAAATTAAAATACC
AATACAATGTTCCGGTGAAACTCTTCCAAAGGAATTTTATGTTTGATTTTTGCAAGGCATGCCTTTAAGCCGTGTCCGGA
TATGTGCGAGTAAGACTGAGCCTGGTTCATCTCATGTAAATACTTGAAAACAGATTTTATGCTTAGTAGCTGTCTGCTGC
CTTTGAATTGTGTATTTACATCACTTTTGCCTCATTGTACAAGGTTGCAATAGGAATTTGTTATGGAAAGCCATGGGCTG
GGAAGAGGAGGCCGTGGGTATAATGCATTTGGATTGAGTAGTGGCGGCAAAGCCGGTCAACCCAATGAAAGTGAAGAGTC
ACAAGCAGGAAGAGGTGGTTTGCTGTTTCCAGGGCGTGGGAGCATTGGAAGAGGGGCATCTTTACTTCGGGGAATGAGTC
GACCAGGTCCATCTGTAGGGCGTGGGACACTTTTCCAAAAATTACTGGGGCCTTCTGCTGGAGACAAAAGTGAAAGCTCA
TCAAGAATAAAAGCTGATGTGTCTGATGGCCGTAGCAGTAACACAGAGTCAATTTCTGAAGATTTGTCGAGTGGAACTGC
ACTCACATCAAATGAGTCATCAAGTCGTAATCGAGCCAAAACAGATTCAGGATTTCATCAAACTCACTCGAGTGGAGGAA
TCACTGGTGCCAGCGGTTCCGTTCAAGTAAGACCACCACCACCAGCATTCCGACTCATGCAGACAGCATTCAGTTCAGTC
TCGCTTGGCAGAGGTTTCAACGTGCCCAGCTTAGGAAGAGGTCTTCGTATGTCTGGTGCAAAAGCACCTATTGGAGTCAC
TACATCAGTTGGTCCAAAATCGACGGGCACGTTGTTTGAAGGTGGTGTTCAGACTCCAAGACAAACAGAAACTGGTCGGT
TTGAGGGTCCAAAAGAGAGTGAAACAGTTGTCCCAAGTCCACAGAGCTCTAAATCAGGAGAGACTGTTGAACCTATCCAA
ACCCTGCCTGCATCTAAAACTAGGTCCCCAGAAATGAGGCAGCCGAGAATGGGTTTGTTGCCTGGGAGGAATATGTCTTC
TGGTAGAGGTGTGACACTCCCTCATCACAAATTAGAACCGACTTTGGGAAGTGCATCAATAAGAAGTCTATCTACTTCAG
AAGCATCGGCTACTCATGAAAGGCCAATGAAACATGATCCACCTGTTATGATGGAAAGGGCAGTCACTGAGTATCATGGC
AATAGTGGTGTGGAAATCCCAACACGTGTCAACTGTGTTCGTGTAAACTGCAGGAACCCTGTCATTTATCAATATCATGT
CACTTTCAAACCAGATGTTGATAGTAGACAACTTCGGTTTTCCTTGATCAACCAACACAGGGAGTTCATAGGACAGAAAG
CGTTTGATGGTGCATTGCTGTATCTAACTAAAGATATTGGTCAAGACACAACGCTGATTTCAGAAAGAATTACAGATGGT
GCCAAAGTTAGCTTGAGAATTCAAATGACAGCGAAACTGAACCCGGCATCACATAAGTTGATTCCATTTTACAACGTGGT
AATGAATAAGGTCATGAAAATACTCAAACTTTGCCCAGTTGGACGAAACTATTATGATCCAGCATGCCCCAAGTCTGTTG
AGAAATACAGGCTTGACTTATGGCCTGGATATGTCACTGCTGTGAGGGAAATGGAAGGCGGCCTATTCTTGAATATAGAC
TGCTCTTTTAAGGTTCTTCGAAATGAAACAGTACTGCAACAGATGCAAGAATTGTATAGAACTTGCAAAGCTGACTTTCG
CGCAAGTTGCATTAAGGAATTGGTTGGAGTGACAGTGCTGACACGCTACAACAATAATAACTATCGCATTGATGATATTG
ACTGGTCGCTTTCTCCAATGAGCACATTCACAACCTCAAAAGGTGAAACCATCAGTTTCTTTGACTACTATAAAAATCAA
TATGGAATTGAAATAAAGGATCTTAAGCAGCCAATGCTGATCAACCGGCCTAAAAAGGCGACTCAGAGACATACCAGAGC
TGAGAAGGGTGAGGAGCGTGTGCTTTCTCTAGTACCGGAGTTATGCTGTTTGACGGGAATTAGTGATAAGCTCGCTGGAG
ATTTTTCAGCCATGAAAGACATTGGAGCAATCACTAGAATGGGTCCTGTTGACAGAAATCTGCATGTTGAGCAGTTTTTG
AAAAGAGTCAATTCATCTACTGAAGCAAGAGGAGAGCTGTTGAAATGGGGATTAGAACTTGAATCCACTTGTGCTGCTGT
CAAGTCTCGGGTTCTACCTGGAGAGAAAGTCATGGTTGGTGGAAACAAGGTATTCCAGTGCAATGAGCAAGTGGACTTTT
TCCGTGACATATCCCGTGCTCCAATGTTGAGATCGGTCCCATTAAAGAATTGGATAATCGTGTTCACTTCTAGAGATCAG
TCCAAGGCAAGAGATTTTGCAACCAAATTTAATGAAGTCTCGAGGTGTATTGGAATGCAAGTACATCCACCTGAGTTCAT
TGAACTGAAAAGCGATCGTAGTGATGAATTCAAGCACGCAATTTCAGGAAACATAAACAATAACCTTCAACTGGTTGTTT
GTATCTTTCCTACTCTTCGTGAAGATCGGTACAATGCGGTAAAAAGACTGTGTTGTGTTGAAAAGCCTGTACCCTCGCAA
GTCATAATATCTAAAACTTTGCCGAAAGATGCCAAGATGGGCAAGTTTCGTTCTGTAACGATGAAAATCGCTCTTCAAGT
CAACTGTAAGTTAGGTGGTGAGCTTTGGGCTGTCTCAATTCCACTTGATGGACTCATGGTTTGCGGGATTGATGTCTACC
ATGAAGGGAAAGGAGGCAAAGGAAGATCTGTGGCTGCGTTTGTTGCGAGCATGAATAAGTTAGTTACAAGATGGCACTCT
CGAGCAATACAGCAACAATTGCCCAATCAAGAAATCATTGATGGCTTGAAGCAATGCTTTTGTGATGCATTAACAAAGTA
TTACGAGTCAAACCATAAGTTACCTGATCGTATCGTAATCTATCGTGATGGAGTTGGAGATGGTCAATTGTCTGTCGTGG
CAGAGCATGAGGTACAGCAATTGAAGGATGCCGTGAAAATGTTCAATGAAATAGCAGGTGGTGTATACGCACCTAAATTT
ACATTTGTGGTGGTTTCAAAGAGGATTAATATTCGTTATTTCAAGAGAGATGGAAATGGTCTTGTCAACCCCCCACCAGG
AGCGGTGTTGGATCATACCGCTACGAGACCAAATTGGCCAGACTTCTATCTGGTGTCTCAGCATGTTCGCCAGGGTACTG
TTTCACCAACGCATTATATTGTTGTTGACGGTTCCGAGAATATGAAACCTGATCACTTGCAACGGCTTACCTACAAGCTC
ACCCATATGTATTATAACTGGCCTGGTACAGTCAGAGTGCCTGCACCATGTCAGTATGCTCACAAATTGGCTTATCTCAT
TGGTGAGAATATACGAATGGAACCCGCTCAGGATTTGTGCGACCGTTTGTTTTACCTATAAACGTTTTCACTGGTCCACA
GAGATGGTATAAACCTAATTATGAGGTATTTTTAGAATTTGAAATTTTTGACAAATTCCTACAGGTTGCACTGGATAGTT
TTATAATTGCACTCCCTTGACATTCAGTCCGTGGAAGGCTGAGTTTGGATTCACAGCTCGACCCTCCCCGAGTGGGGGGA
TGTGTCGTAGGAAGAGCGTCATGGTGCAAAACTACCCCTCAAACCTCATCTTGCTGAACTGGATCAAAGCATCCCCTGTC
CACTACTTTCCCCCTTTTTACAAGCATTTTTTATTTTTTAATCAAGACATTTTTGATCATTGCAAATGACGCCATCTGTA
AAGTGGACCTGTTCCTTTGATTTGCACACGCTTCAATGTGTGCATGAGCTATGTTATCATTACTTTGATTATGATGGTTT
TGTCCTAAAGCTGTGTTTGCAGTTCCATTTATCTCATAGCCTTGTGGATTCAGTTCCGATGACAATGGTCGGAAATCATA
GTTCCACTTTGGAAAGTTTTACTTTATTTGGTGAGTCAAATTCGTTATGACTTTAGAAAATTGTGCATGATTGAGTTTCG
TTTCAAACTGCTCCCTGTTATGCTGCATAGTCTGGATTTTGTGCTCTTTTAGAATGTGTATTAATGTCAAAATCTGGACA
AAAAGTTGAGCAACCTTTGGCAATCAGTTAACTACCTGTGTATGACAGGATCGGAAATATAGGACGTATATTTATTCTGT
CCTTCAATTTCGCTGCATATACACAGATAATGATGATAGGCCTACAGACGACATGTGCTGCGAATTTCGTTCCTTTTGTG
AGTTGAATACACGCACAAACAGCGGCTATTTGGGACGTGATCCGTAGGACAAAACTATCTTTCCCTTTGGCCTATCTGTA
TCGGTGTGCGATATAGCATAACAGCCGAGGCGAGTTTGGTATGTTTCTGCGTCTGATGGTTATCAGCAGGGACTAATGTT
GATGGTGGTGGGGTCTTAAGACAGAAAGACACCAAACTCTGTGAAGTTTGGATGAGGCTTGTATGGCGGAAGACATGAGG
TTGTACTTTGAGTTTCCCACTGACTGAGGCACTGACCTTATGCTAAAGGTCTAGTCGGCACCGCGAGTATGTTGCTGATG
CTGTCAAGCAATTCCAGGCCATCGCACGACGCGGCAGACATAAAACCCACAAAGCCGAGGCAGTGAGTAGGCCTTCGGTG
TGTACTTAGTGAGTGCGACCATGATAGGTACTATGACTTGCGGCGTTTCACACAGCTCGATAAAAGTGGAGCGATAATTG
ACCGTCATTGTCGATTCCTTGATCGATGATTCAAAAAGATGGAAGAAGCCGTTTTTTATGTCAAGACAAACACGAACAGC
TTTTGCGAGATATGTCTTGCCATTATGCATTCATTAGTCTTGGTATTGTATAGTAGACCTTTGAAGCAGATATGGTTTCC
GGATGAGTATTTCTGGTTTTTACACATCAACGGCTTACGCCAAGTCACCAGCCCAAGCTTCAACAGAATAACGCAGGCAG
ATTGGCCCCTGTCGTGCTAATTTTTCACGTGTTTTTCCAGGAGTTGTGGCCACAGATGCCATAGTCATACAATACAGTTG
TCTAAGACTTTACAGCAGACACTCCTGCGGGAACATGCCGCATCCTGCCAGATGCGATGTACTGATGCGCATCACGCAAT
CTTTGCACGTTGCAACTCCTCATGACTGGTGAAAGCTTGCCGCTCGTTCATTACAATGAAGGTAAAATATACCGCTTTTA
GATTCAGATGTATGCATGTGTACATTTTCTATGTACCTTCCCTTCTCTATAGGCCTGATAACAGCGCCGAGATATCCATC
CTCTCTGACCACTGAGCCAGGTAACAACTCAAGCAACACGAACTCATGTCGTCACGGGAACTTCGTGTTCGTTCGAGGTC
AAGCTGACGCTGAGTACCAGTACCGTACTGTTCTCGAAAATGTATGCAGCGGGATTGACGTGACGTCTTTCCGTGAGGTG
TCGCCTAGGGATTGCGCGGTGATACGGTAATCAGTCTAATCGTAGAACGTAATCGATGATATACTTCGCTTACGGTACAT
TGCGGCCGTTTTGGCAGCCAGTGGTAACAGGCACACGGTTCTTGTCGCCTAATGTGTAAGAGAGTACCGGTACAACGTAT
TTCCGTTCGAATGTTTCGCACCAACTTATGAACGAGATTTCACTTTTAGACGTGTAGCCTAGCCTACAACTTGAAATGCT
CTGTTTCCGCGAATCTTCGTAACTTCCATTTTCGGGGATATCACAACAGAAGCCGAGCTCAAGCAACTAGAAACTACCCA
ATAGGATGAGTCTCGTAAGTTTTTTTTATCTGTTCAACGGGTTCCACCGATACAGTACTTCACGACAGTTGCGAGTAGTG
TTTCAGCGAGTCACCATCTGCGCTGTAGGCCCAAGTGGTTATTAGGCTAACGCTAAGCCTATATCTAGTCTCACCGAGTT
GTTCGAAAATCGTGGCAATCGTATCTTTGAAATTTTATATCGTCAAATGGGAATCACGAAGCACTTTTCGTAACGATACA
TATCTATGGCAAATTTGACAGCTCATGGGGCATAGGCTATCAGAGCGATCGCTTGTGAAAGAAGCAGATACTCACCGCGT
CGGCTTGGTGTAGATACAGGACCGTGATAACAGCGACGAAAAGTAGTTTTGTGT
