# Supplementary material for: Integrin-alpha-6+ Candidate stem cells are responsible for whole body regeneration in the invertebrate chordate Botrylloides diegensis
Source: Nat Commun. 2020 Sep 7;11:4435. doi: 10.1038/s41467-020-18288-w (PMC7477574; doi:10.1038/s41467-020-18288-w)
Supplement: Supplementary file 9 — Supplementary Data 5 [file 41467_2020_18288_MOESM9_ESM.rtf]

Supplementary Data 5. Primer and probe sequences

qPCR primers (B. diegensis):

actin Forward: AACAAGAAATGCAGACCGCC
actin Reverse: GGCCGATTCCATACCCAAGA
PCR product length:	146

cyclin b Forward: CCCCGCATGAGAACCATACTT
cyclin b Reverse: GCAAGCTGTAACTTGGTGCG
PCR product length:	146

Integrin-alpha-6 Forward: GGGATAGTAGTGCTGACGGC
Integrin-alpha-6 Reverse: CAGCGATGTCGGGATAACCA
PCR product length:	156

Pou3 Forward: CGATCTGTCGATTCGAGGCT
Pou3 Reverse:GGTTGCGTTGTCTAATGGCG
PCR product length:	146

Vasa Forward: ACTGTTGGATCTCAGCCGTG
Vasa Reverse:	TCTGGCTCAAAGCCCATGTC
PCR product length:	153

Notch 1 Forward: GAACTCACTGGAAGGTCGCA
Notch1 Reverse: ACACTCCACCAACAGCGATT
PCR product length:	145

Notch2 Forward: TGCATGAGTAGCCTGGAACG
Notch2 Reverse: AGGATCTTGTACGCCATCAGC
PCR product length:	158

Hes1 Forward: ACTTGGATACGGGTGTGTGC
Hes1 Reverse: GTCAACGTGTTTCCGCCATC
PCR product length:	155

Frizzled 5/8 Forward:	GCGGAACAAAACCTGCAAGA
Frizzled 5/8 Reverse:	TTTCTTCCAAGGCAGGGCAA
PCR product length:	156


Dishevelled Forward: AACCTCGCTATCGCCACAAA
Dishevelled Reverse:	TGATGCATGTTGCCATTCGT
PCR product length:	154

Beta catenin Forward: AAGATGACGTAGAGCGCACA
Beta catenin Reverse: GTGCTGCTGGGATTCTGAGT
PCR product length:	158


piwi1 Forward: ACCAGGAAGCGACTGACTTG
piwi1 Reverse:GGAGCTCGTTGTCCCAAGAA
PCR product length:	156

Piwi2 Forward: TAGGGCGTGGGACACTTTTC
Piwi2 Reverse: GAGTGCAGTTCCACTCGACA
PCR product length:	149


FISH probe cloning primers and probe sequences (B. diegensis):

Integrin alpha 6 Forward: TGGCGTTGTAATCGTCGTGA
Integrin alpha 6 Reverse:  TGAGCAGTGCATTTCCCGAT
PCR product length:	764
CTTGGCCGCTATAGCCATGAGGGATATGGATACTGCCAGGCTGGAGCCTCGGTGAATTTTGCTGATGTTCCTG
CTAGCGATGATGGAGAATCGTATGTCTTGATTGGAGCCCCGGGGAGTATCCACTGGAGTGGCGCTGTTCTTGCTACAAGA
AAGGGAGGTGATTTTGGTCTGTCGGTTGAGAAAGTATGGTCTGATAAGGATCTCACCATGACAAATTATCAAATGGGCTC
TTCTGTTCTGGCTGGTTTTATCTACAGAGAAGATGCTGTAAATTTTGTCACTGGAGCACCCGGCGCTAACACTACAGGTG
CTGTGTATATACTTGAAAAAACGACACCCGACGTTACTAATTCTGATGGAGATAGTTACCTGAGAATTGTCGAGACGGTA
AATGGTGATAAGGTGGCTTCCAGATTTGGACACGACATACTTTTGTTAGATGTTACAGGAGATGGGAAACTTGACCTGAT
CATCGGTGCACCTCAGTTTTATGATAGAAATGATCAAGTTGGTGGGGCTGTCTATGTTTACGTGAATAAAGGTTTGTCAA
CCATTGGTCCATCACCTACTCAGAGGTTGTTTGGTAACATTGACTCCTACTTTGGGATGGCTGTAGCAAGTGCTGGAGAT
GTTAATATGGATGGAGTTAATGACATTGCAATCGGTGCTCCGGGTGGAAATAAATGGACTGGTGTTGTGTATATCTATCA
TGGGGATAGTAGTGCTGACGGC


Pou3 Forward: ACACCGATGCAAAAGTTGCC
Pou3 Reverse: AACAAAATCGTCGGGCTCCT
PCR product length: 865
ACACCGATGCAAAAGTTGCCGTTT CGAAACTTCTGCACGGCGGCAGAACGCGGATTTTCAAATGAAACGCAATTTGAACAAAAGTTTACATCCGACGAATCTAT GATAGGCACAGAATCGTCTGACGACATGCGAATTTTCGCAAACGTTTTCAAAGCTCGTAGAATAAAACTCGGTTTCACTC AACATGACGTTGGGTTAGATTTAAAAAAGTTTCAAGGGTCCGCATTCAGTCAA
ACTACGATCTGTCGATTCGAGGCTGGG GGTTTAAGCATCAAAAACATGAATAGGTTAAAACCTCTATTAACAATGTGGCTACGACACAACGATACCGAGCATATTTC AACCTTGAGGGATACTGATCGATCGCCATTAGACAACGCAACCACTCGAAAAAGAAAGAAACGAACGTGTATAGAACCCC AAACTAAACTTGCTCTTGAAGAAAAATTTAGGAATGATCAGAAACCAACAACAGTTCAAATTGCTAAAATTGCAGAAGAA CTGTCATTAGATAAAGAAGTGGTGCGAATTTGGTTTTGTAACAGGAGGCAGAAAGAGAAAAAGGCAACAGTTGAAATTGT GCAGAGGGATGTGGCATAATTGAAACGCATTTTTTCTTGCTCAGTGGAATTTAACTTTTTCATACTTCATACTTCTTAAT ATTATAATATACTCTTTCAAAATGCATGAATTGGGATTGCGTACATCTATATTGAAACCAGTCTAGTATGCGTATGACTT CCAATCGTAGGTCTACAGCTCTTGCGACCGTATTACCCATGAAGCAAACAAACCTCTTGCATGGGGACCCGCGTCACGTG GTTTAGCAATAAAGGTTCCCAAGGAGCCC
GACGATTTTGTT


Vasa Forward: TGTCCCGTGACTGTCCTAGT
Vasa Reverse:	TGCAAACTTGCGAGCCTCTA
PCR product length:	833
TGTCCCGTGACTGTCCTAGTG
GAGGAGGTGGCGGCGGTGGCTCACGAGCTTGCTTCAAGTGTGGAGAGGAAGGCCACATGTCCCGAGAATGTCCCAGTGGA
GGTGGTGGATCTCGCTCCAAGGGTTGTTTTAAATGTGGAGAGGAGGGCCACATGTCTCGCGACTGCCCCAATGGAGGGGG
CGGTGGTGGCGGAGGCTCACGAGCTTGCTATAAATGTGGCGAAGAGGGCCATATGTCCCGCGAATGTCCCAATGGTGGAG
ACTCTGGTGGCTTTGGAAGAAAAGGTGGTGGAGATGGTGAAAGAAGCAATGCCTGCTTCAAGTGTGGTGAAGAGGGGCAT
TTTTCAAGGGAGTGTCCCAAGGCTGGTGAAAGTGGTGATGCAGACAGGCCTGAACCGTACATACCACCACCGCCTCCTGA
GGATGAAGAGGCTATCTACGCTTCTACTCAAACTGGCATCAACTTCAATAAATACGATGCCATTCCAGTGGAAGTCACAG
GAGACAATCCTCCAGATGGAATTGTTACATTCGATGATGCACAGCTGCCTGAGACAGTGCGTGAAAATGTCAGAAAGGCT
AAATATACGAAGCCGACTCCTGTCCAAAAATATAGCATATCAATAATCAACGGGGATAGGGATTTAATGGCCTGTGCCCA
AACTGGGTCTGGTAAGACTGCCGCGTTTCTGCTGCCTGTTTTGGCTGGGATATTCAAGAATGGGTTGAAGAGTGGTGACT
CTTTTTCTGGCAAACAGACCCCACAAGCCATCATTGTAGGGCCTACGAGAGAATTGGTGTATCAAATCTTTCTAGAGGCT
CGCAAGTTTGCA


Histone 3 Forward:	GCGGTCGTCTACACACTTCG
Histone 3Reverse:	CACAAGGATTGGGTGGCTCT
PCR product length:	565
GCGGTCGTCTACACACTTCGTTCAGCAGCAGCAGCAGCAGCAGCAGCAACAGCGGCAGTA
GCGGCATCGGCGTCAGCATCAGCGAAGGCGAATAGAAAAAACATGGCTCGTACAAAACAGACTGCTCGCAAGAGCACAGG
AGGCAAGGCTCCACGAAAGCAGCTCGCCACCAAGGCGGCCAGGAAGAGCGCGCCAGCCACCGGCGGCGTCAAGAAGCCTC
ACCGTTACAGGCCGGGCACGGTGGCGCTCCGTGAGATCAGACGCTACCAAAAGTCGACCGAGCTGCTCATACGCAAGCTT
CCGTTCCAGCGACTGGTGCGCGAGATCGCCCAGGACTTCAAGACCGACCTTCGCTTCCAGAGCAGCTCCGTGATGGCGTT
GCAAGAAGCCAGCGAGGCCTACCTCGTGGGTCTGTTCGAAGACACCAACCTGTGCGCGATCCACGCCAAGCGCGTCACCA
TCATGCCGAAAGACATCCAACTTGCCCGACGCATTCGGGGCGAGAGGGCTTGAGCGAGTCCGACTCTACAGAAACGGCTC
TTTTCAGAGCCACCCAATCCTTGTG


Notch1 Forward: AGGCACTTGCATTGACGGTA
Notch1 Reverse: CCTTCACACCTTGGTCCCTC
PCR product length:	792
AGGCACTTGCATTGACGGTATTAACTCTTTCACTTGTTCCTGCCGAAGTGGATACGCAGGAACGCATTGTGAGGAGGAGA TCGACGTGTGTACGTCGGTAGTTTGTGAAAATGGTGGGACTTGTTTTGTTGAAGGAGGAATGCCAAGATGTGCTTGTACT ACGGGCTATGAAGGGACATTATGTGGGGACCTTGTCGATTTATGCATTGATCCATCTATTTGTCAAAATGGTGGAACTTG TACTCAATCTGGAACTAATATGAAATGTGCTTGCACTGACAGCTACACTGGAACTTTCTGTGATGTGCCAAAACTTACTT GTTCACAAGCAGCTGCTGCAAAGGGAGTGAGTGAGCAGAATCTATGTCAAAACGGAGGGACGTGTGAAGACTCAGCAGAT GCTCACCATTGCAATTGTGTGGCTGGTTTTACGGGATCTTATTGTGAGACGAATATTGATGAATGTGTTTCAATGCCATG TAAAAACGGTGGTACTTGCAACGATGGAATCAATGGCTATTCATGTGATTGTGTCGTGGGTTACACCGGGACCAATTGCC AGACTAATGTTGATGAATGTGAATCACAACCTTGCCAAAATGGTGGAACTTGTATCGATATGGTCAACGGATTTCAATGT TCATGTCTGGCAGGAACGTCAGGCACCTTGTGCGAAGTGAATCACAATGATTGCGATGACGATTCGTGCTACCATGGCGG TACTTGCATTGATAGAATAAATGGATTTGATTGTAATTGCCTTGATGGTTTCGAGGGACCAAGGTGTGAAGG
TGATGTGA


Notch2 Forward:  AAGTGCCTCCGTTCAAGCAA
Notch2 Reverse: CCCTGGTTACACCGGCATTA
PCR product length:	653
AAGTGCCTCCGTTCAAGCAAGAGCTGCTGGTGCAGTCATTTGGATTATCCGTACAAGTT

GAAGATGAGTATCCGTCTTTGCAAGTACACACAAAACTGTTAACATAATCCTCGCAAGTT

CCTCCATTTTGACAAGGATTGCTGGCACATTCATCAATTTCATATTGGCAATTGACGCCG

TCATATCCAGGCTTGCAAAGACATTTATACGTCCCGATGTCATCGATGCATCTAGCTCCA

TTAACGCACGGATCTGGTGAACAGTCATCGATATTAGTCTCACAGTTTGCACCGTTAAAA

CCAGTAGAGCAAGTGCATGAGTAGCCTGGAACGCGGTTGGTGCAATGCCCTCCATTTTGA

CAAGGATTTTGAAGACACTCGTTGATATCTTTAGAACAAGTTTCGCCTTCAAATCCTTCA

TCACAAGCACAAGTATATGTCAAATAATCAGCTGATGGCGTACAAGATCCTCCATTATTG

CATGGATTCGGTTCGCACGGTGACAGTTCAGTTTCGCAGTTTGTTCCTGTATACTTAGCC

GGACAGTCGCAAGTGTAGACGTTCACCCCGCTAGTACAAGTTCCACCGTTTAGACAAGGT

CGAGTAGCGCATTCATCAATATCCACATCACAAGT
AATGCCGGTGTAACCAGGG
