# Supplementary material for: Integrin-alpha-6+ Candidate stem cells are responsible for whole body regeneration in the invertebrate chordate Botrylloides diegensis
Source: Nat Commun. 2020 Sep 7;11:4435. doi: 10.1038/s41467-020-18288-w (PMC7477574; doi:10.1038/s41467-020-18288-w)
Supplement: Supplementary file 10 — Supplementary Data 6 [file 41467_2020_18288_MOESM10_ESM.rtf]

Supplementary Data 6. pou3 protein sequences

Pou3-phylogenetic analysis – protein sequences
this data was used to generate the phylogeny presented in Supplementary Figure 1A. 


Botryllus schlosseri Class 4 POU (POU_c4_Bsch):
FESLTLSHNNMVALKPILTTWLELAEEEYRRKMEQSGLAEKKRKRTSIAAPEKRSLEAYFLVQPRPSSEKIAAIAEKLDLKKNVVRVWFCNQRQKQKRMKFSAFNGENGGM

Botryllus schlosseri Class 2 POU (POU_c2_Bsch):
MLQYQQHRMFEDRRHSGGEFIHARPPSPGMHPGISQSPXHQSDYEETSSKRQRYEDEASELESLERFAKDFKQRRIKMGFTQGDVGVAMGRFYGNDFSQTTISRFEALNLSVKNMGKLKPLLERWLIDVDRAISTGERSEGRPVLSQPMMAMNSQSCAGRKRKKRTSIPTEGKSRLEDAFLKNPKPTTEEIGKFSEDLNMDREVVRVWFCNRRQKQKRIATQQQRYVGEQHSPSSPAGFNEPHSVEGQRSPISSEDENYGSHHLSDRPPVHQAIEHSEMGTLPPQLPPRPGIIQLKLSESHQMQFGRPPNYSSHSSLMGGNSAI

Botryllus schlosseri Class 3 POU (POU_c3_Bsch):
VEFYEMLSQVPNGELAYGSPLQDGCGKYSSHSEVGKCRNKTRIHENPHMPNHSPVMSYSTPGLSTYACLDSQPPIRSDTSQEQESFTKISDEHYRPYPNGYQFSNHCYQFNQSGYHRALPIPSLQEQLYSVHDSPRQRIPSRSPHTSQVTEDFVIKESPAYTNDANVQSWHSFVHSAREISESSRLNTTSNAGCYPIANSVCASDKAGCVYSYQNQPGQYPYCYSRNYYPASTNLQNRTWNPRPIDLSLKQDHCDGYGEMDYQPTHFTSYSPLRIDERNLLSDERTLNKLPCDDMSLNGWTEEDMRQFSKVFKHRRTKLGYTQSDVGTSLGELYGSVFSQTTICRFEAQQLSLKNMCKLRPLLSRWLQHKDNKHETLTPDIDQIDSENGPGRKRKKRTSIEAEVKAVLEKHFKLKPKPMTQEIVSIAEQLSLEKEVVRIWFCNRRQKEKKVNEQVMRSQNPT

Botryllus tuberatus Class 2 POU (POU_c2_Btub):
LQGKEQKQKKLDYSQEDDRFAPAPGFHERHFVRQPSDFDVDPRQIRPDFAIRHSMLSHPVLLDNRRCSEENMSPPGPESPTVTPANMARHIPQSFSSDDQCDYDEIPKKRRMYDDEASELEDLEQFAKDFKQKRIKMGFTQGDVGVAMGKFYGNDFSQTTISRFEALNLSVKNMCKLKPLLERWLIDVDRAISDRGEGGRLAISQPVIPMHPQQCTGRKRKKRTSIPTEGKTRLEDAFKKNPKPTTEEICKFSENLKMDREVVRVWF

Botrylloides diegensis Class 3 POU (POU_c3_Bdie):
NNFYAHVSLTNENGRSVIAQGNECSGKQSPEYKEYETPIKELTYMSLHPRTVTSNGYYSQSSTFGENFRHFPTQSPYTGHDHNPGYNYNSYPPTLIPADCLQGSLQSRHSFNSTFAPESYASEQNSDHPRISRSAPPNVTITECSDTHYNANSVKCFSDRHSEYSYPHLPSPANMGVVCKREITTPSPQLRHEISSSWRGQELCPPSYLHQDTPQYRYSYQANYWPLSPANSTSCSYQKASSNRFVKQERFQDYQTNTPMQKLPFRNFCTAAERGFSNETQFEQKFTSDESMIGTESSDDMRIFANVFKARRIKLGFTQHDVGLDLKKFQGSAFSQTTICRFEAGGLSIKNMNRLKPLLTMWLRHNDTEHISTLRDTDRSPLDNATTRKRKKRTCIEPQTKLALEEKFRNDQKPTTVQIAKIAEELSLDKEVVRIWFCNRRQKEKKATVEIVQRDVA
